# Biomimetic Analogues of the Desferrioxamine E Siderophore for PET Imaging of Invasive Aspergillosis: Targeting Properties and Species Specificity

**DOI:** 10.1021/acs.jmedchem.4c00887

**Published:** 2024-06-22

**Authors:** Andrzej Mular, Isabella Hubmann, Milos Petrik, Katerina Bendova, Barbora Neuzilova, Mario Aguiar, Patricia Caballero, Abraham Shanzer, Henryk Kozłowski, Hubertus Haas, Clemens Decristoforo, Elzbieta Gumienna-Kontecka

**Affiliations:** † Faculty of Chemistry, University of Wrocław, 50-383 Wrocław, Poland; ‡ Department of Nuclear Medicine, Medical University Innsbruck, A-6020 Innsbruck, Austria; § Institute of Molecular and Translational Medicine, Faculty of Medicine and Dentistry and Czech Advanced Technology and Research Institute, Palacky University, 77900 Olomouc, Czech Republic; ∥ Institute of Molecular Biology, Biocenter, Medical University Innsbruck, A-6020 Innsbruck, Austria; ⊥ Department of Organic Chemistry, The Weizmann Institute of Science, Rehovot 7610001, Israel; # Public Higher Medical Professional School in Opole, Katowicka 68, 45-060 Opole, Poland

## Abstract

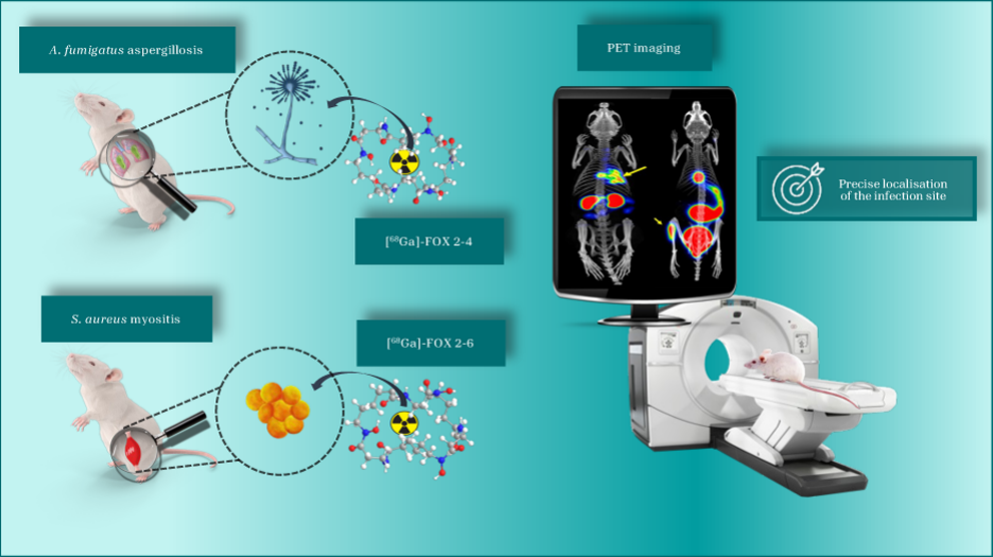

The pathogenic fungus *Aspergillus fumigatus* utilizes a cyclic ferrioxamine E (FOXE) siderophore to acquire iron from the host. Biomimetic FOXE analogues were labeled with gallium-68 for molecular imaging with PET. [^68^Ga]Ga(III)-FOXE analogues were internalized in *A. fumigatus* cells via Sit1. Uptake of [^68^Ga]Ga(III)-FOX 2–5, the most structurally alike analogue to FOXE, was high by both *A. fumigatus* and bacterial *Staphylococcus aureus*. However, altering the ring size provoked species-specific uptake between these two microbes: ring size shortening by one methylene unit (FOX 2–4) increased uptake by *A. fumigatus* compared to that by *S. aureus*, whereas lengthening the ring (FOX 2–6 and 3–5) had the opposite effect. These results were consistent both *in vitro* and *in vivo*, including PET imaging in infection models. Overall, this study provided valuable structural insights into the specificity of siderophore uptake and, for the first time, opened up ways for selective targeting and imaging of microbial pathogens by siderophore derivatization.

## 1 Introduction

*Aspergillus fumigatus*, a ubiquitous fungus, has became the most common mold pathogen of humans.^1^ The mortality of invasive aspergillosis, the most serious disease caused by *A. fumigatus*, reaches up to 100% in immunocompromised patients.^2^ Current limitations in diagnostic and therapeutic options necessitate innovative approaches.

The severity of *A. fumigatus* infections arises from its ability to adapt to diverse, often hostile environments,^3^ including nutrient-poor niches.^4^ Inhaled spores must germinate in an environment with the lowest free iron content on Earth.^5^ Growth there is enabled by the use of siderophores, low-molecular weight organic compounds with a high affinity for ferric ions that are dedicated to microbial iron acquisition and storage.^6−8^ The use of siderophores is highly conserved in the fungal kingdom and is considered an important virulence factor; fungal mutants unable to utilize siderophores are unable to efficiently colonize the host organism and cause disease.^9−11^*A. fumigatus* is known to produce four hydroxamate-type siderophores:^12,13^ triacetylfusarinine C (TAFC) and fusarinine C (FsC) as major secreted siderophores for iron acquisition and ferricrocin and hydroxyferricrocin for intracellular handling.^12^ Recently, ferricrocin was also found to play a role in iron acquisition.^13^ In addition, *A. fumigatus* utilizes ferrioxamine B (FOB) and ferrioxamine E (FOXE, DFO E, nocardamine) as xenosiderophores.^2,14−17^

In the fungal kingdom, cellular siderophore uptake is mediated by the so-called siderophore iron transporters (SITs), a subfamily of the major facilitator superfamily.^18^ Recent studies revealed that *A. fumigatus* utilizes four SITs: Sit1, Sit2, MirB, and MirD.^19−21^ Sit1 was shown to be the exclusive transporter for the bacterial FOB, FOXE, and ferrioxamine G;^19,21^ the transport efficacy of linear ferrioxamines is impacted by their charge and consequently the environmental pH.^15^

**Figure 1:**
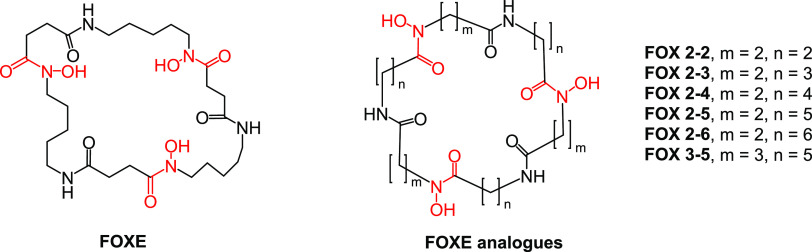
FOXE and its FOX analogues were investigated in this work. Iron-chelating hydroxamic acid groups marked in red.

FOXE (Figure 1) is a cyclic, trihydroxamate siderophore native for bacterial species such as *Nocardia*,^22^*Pseudomonas stutzeri*,^23^*Enterobacter agglomerans*,^24^ and those from the *Streptomyces* genus.^25^ This siderophore is a common target for siderophore piracy^2,7,16^ and is recognized as a xenosiderophore for iron uptake by bacterial and fungal species such as *Pseudomonas aeruginosa*,^26^*Staphylococcus aureus*,^27^ and *A. fumigatus*.^19,28^

Siderophores are gaining increasing attention within the scientific community due to their potential as targets for pharmaceutical interventions. A promising approach involves leveraging these compounds in the fight against invading microbes as diagnostic markers or antimicrobial therapeutics.^11,29−33^ For example, it is possible to deliver radioactive probes by siderophores into fungal cells, facilitating precise and specific diagnosis. This was previously successfully implemented for nuclear imaging of *A. fumigatus* in rodent infection models using ^68^Ga(III) complexes of TAFC and FOXE.^34−36^ FOXE was successfully labeled with Ga-68 with high radiochemical purity and showed high uptake by *A. fumigatus*, favorable pharmacokinetics, highly selective accumulation in infected lung tissue, and good correlation with the severity of the disease in rat infection models.^34,37^

In the next step, applying a biomimetic strategy,^11,38−41^ we have designed and synthesized a series of six analogues of natural FOXE (Figure 1).^42^ Their synthesis, solution chemistry, and coordination properties have been reported in our previous work together with the biological activity in *S. aureus* cultures^42^ (for the summary of key information regarding FOX ligand and complex characterization, please refer to the Supporting Information supplemented to this work). The iron binding cavities of the discussed FOXE analogues differ from the natural FOXE with regard to the cycle length and position of the hydroxamic group in relation to the amide group. The FOX *m*–*n* numbering (with *m*–*n* from 2–2 to 2–6 and 3–5) describes the carbon atom quantity between the hydroxamic–amide–hydroxamic groups, −N(OH)CO–(CH_2_)*_m_*–NHCO––(CH_2_)*_n_*–N(OH)CO–. FOX 2–2 to 2–6 analogues have the same ethyl spacing group between hydroxamic and amide groups (*m* = 2, like in natural FOXE), while FOX 3–5 has a propyl group spacing them. The hydroxamic acids are positioned retro in relation to natural FOXE. FOX 2–5 is therefore the most similar compound compared to FOXE, with the same atom composition and molecular mass, differing only in retro positioning of the hydroxamic groups. FOX 2–6 and FOX 3–5 also exhibit the same composition and molecular mass but differ in the arrangement of hydroxamic and amide groups; both analogues form larger cycles than FOXE. FOX 2–2, 2–3, and 2–4 are smaller than FOXE. All the structural changes were implemented in order to evaluate the role of the cycle size and composition in the ferric-siderophore complex formation and its recognition by the corresponding transporters.

The applied structural changes influenced the physicochemical properties of the analogues as well as their biological activity (for the summary of key information, please refer to the Supporting Information supported to this work). Four of six designed analogues retained the biological activity of their natural counterpart and presented high potential during *S. aureus* in vitro assays as stable carrier agents for Ga(III) ions.^42^

In this work, we analyzed the biological activity and SIT specificity of these FOXE derivatives in *A. fumigatus*. Remarkably, some of these analogues exhibited species specificity, in contrast to native FOXE. The proper understanding of the molecular interactions between FOXE analogues and the transporters is crucial for the full utilization of the siderophore system for selective diagnosis and treatment of microbial infections. Here, we present the first results of PET imaging in rodent infection models with ^68^Ga-FOXE analogue complexes as a promising diagnostic tool.

## 2 Results

### 2.1 In Vitro Characterization

#### 2.1.1 Radiolabeling of Siderophores

Radiolabeling of siderophores showed the same results as described previously.^42,43^^68^Ga-labeled FOX 2–5 was obtained in quantitative yields with high radiochemical purity (>95%), whereas all other compounds were purified by solid-phase extraction.

#### 2.1.2 Growth Promotion Assay

**Figure 2:**
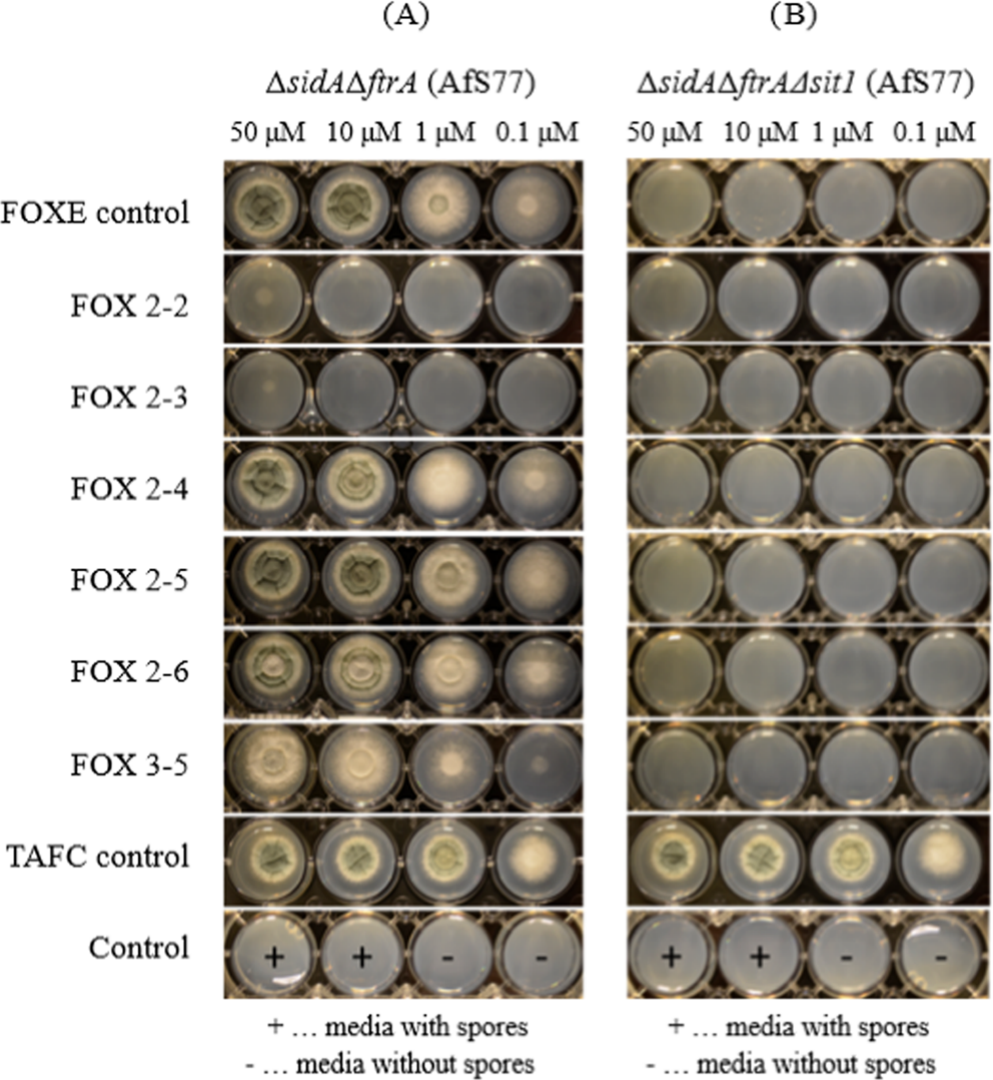
Growth promotion analysis of FOX analogues. 10^4^ conidia of *A. fumigatus* mutant strains Δ*sidA*Δ*ftrA* (A) and Δ*sidA*Δ*ftrA*Δ*sit1* (B) were point-inoculated for every condition. All natural and artificial siderophores were tested in the ferri-form in the concentrations 0.1, 1.0, 10, and 50 μM. As controls, TAFC, which is taken up in a Sit1-independent manner, and FOXE were included. Moreover, controls without siderophore supplementation with (+) or without (−) inoculation of conidia were included. Greenish coloration of the fungal colonies indicates asexual sporulation. Pictures were taken after 48 h of incubation at 37 °C.

In order to investigate whether *A. fumigatus* is able to utilize the artificial FOX analogues in the iron form as an iron source, growth promotion assays were performed (Figure 2) using two *A. fumigatus* mutants. The mutant strain Δ*sidA*Δ*ftrA* is able to grow only in the presence of utilizable siderophores or ferrous iron concentration >3 mM.^10,44^ The second mutant, Δ*sidA*Δ*ftrA*Δ*sit1*, additionally lacks the siderophore transporter Sit1, which exclusively mediates the uptake of ferrioxamine-type siderophores and is additionally involved in the uptake of ferrichrome-type and coprogen-type siderophores but not of FsC or TAFC.^19,20^ For growth promotion analysis, the mutants were spotted on iron-lacking media supplemented with different concentrations of siderophores. Control experiments confirmed the expected growth pattern: growth of Δ*sidA*Δ*ftrA* but not of Δ*sidA*Δ*ftrA*Δ*sit1* was promoted by supplementation with ferric FOXE; both mutant strains were able to grow in the presence of TAFC as uptake of this siderophore depends on MirB but not on Sit1; both mutant strains lacked growth without siderophore supplementation, and uninoculated media also lacked fungal growth. Supplementation with ferric FOX 2–2 or FOX 2–3 showed very limited growth promotion only at the highest tested concentration of 50 μM, and this growth was dependent on the presence of Sit1. In contrast, FOX 2–4, FOX 2–5, FOX 2–6, and FOX 3–5 promoted substantial, Sit1-dependent growth. The degree of growth promotion and sporulation, which is reflected by the greenish coloration of the colonies due to the greenish coloration of mature spores, indicated that FOX 2–5 shows the best utilization by *A. fumigatus* followed by FOX 2–6, FOX 2–4, and FOX 3–5. Taken together, these results suggest that *A. fumigatus* is able to take up the artificial siderophores with various efficacy following the order FOX 2–5 > FOX 2–6 > FOX 2–4 > FOX 3–5, while uptake of FOX 2–2 and FOX 2–3 was negligible. Notably, FOX 2–5 displayed growth promotion similar to natural FOXE. The utilization of iron from all artificial ferrioxamine analogues was mediated by Sit1 like that of natural ferrioxamine-type siderophores.^20^

#### 2.1.3 *In Vitro* Uptake of [^68^Ga]Ga(III)-Siderophores

**Figure 3:**
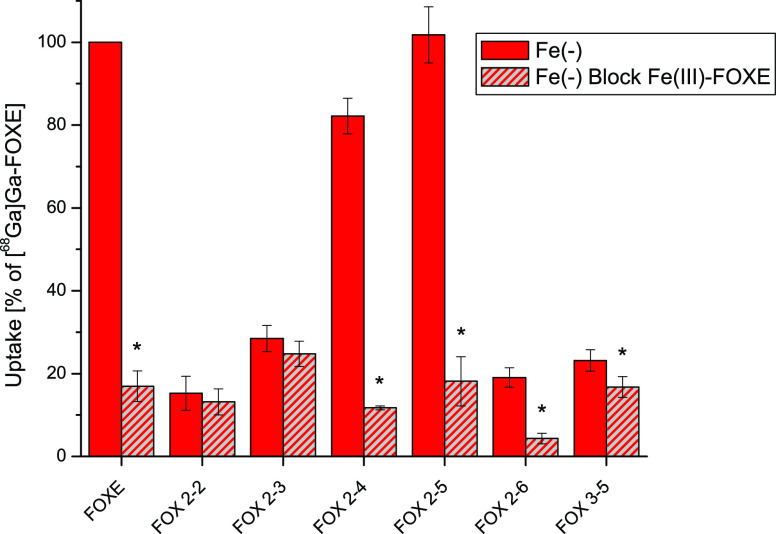
Uptake of ^68^Ga-labeled FOXE analogues by Fe(III)-FOXE-blocked and -unblocked iron-starved (Fe(−)) *A. fumigatus* cultures after 45 min of incubation. * indicates significant difference of blocking compared with nonblocking (mean of three independent experiments, paired *t* test, *p* < 0.01).

To investigate the short-term active transport of ^68^Ga-labeled FOXE analogues by *A. fumigatus*, iron-starved (Fe(−)) cultures were used (Figure 3). As a control, the same cultures were incubated with Fe(III)-FOXE beforehand to saturate and therefore block Sit1-mediated activity. A decrease in uptake upon Fe(III)-FOXE blocking underlines specific uptake by the same transporters recognizing FOXE.

[^68^Ga]Ga(III)-FOX 2–5 showed excellent uptake in Fe(−) cultures with no significant difference compared to [^68^Ga]Ga(III)-FOXE (Figure 3). In agreement with specific uptake, blocking with Fe(III)-FOXE decreased the uptake to about 15%. A similar but slightly lower uptake behavior was found for [^68^Ga]Ga(III)-FOX 2–4. The other compounds, however, revealed a different pattern. [^68^Ga]Ga(III)-FOX 2–3 showed an average uptake of 24% and was not significantly reduced by Fe(III)-FOXE blocking. These findings indicate certain unspecific binding or Sit1-independent uptake of FOX 2–3. Similar values were found for FOX 2–2 and FOX 3–5. [^68^Ga]Ga(III)-FOX 2–6 also showed a low uptake of 16%, which however exceeded that of Fe(III)-FOXE-blocked cultures by fourfold (4%).

It can be clearly seen that [^68^Ga]Ga(III)-FOX 2–5 showed neither significant difference to the standard [^68^Ga]Ga(III)-FOXE in iron-starved cultures nor when blocked with Fe(III)-FOXE, indicating identical recognition by Sit1. [^68^Ga]Ga(III)-FOX 2–4 showed slightly reduced uptake with a highly reduced level in blocking conditions. All other FOX biomimetics revealed much lower uptake values. As shown in Figure 3, the difference between Fe(III)-FOXE-blocked and -unblocked uptake was statistically insignificant for ^68^Ga-labeled FOX 2–2 and FOX 2–3 but statistically significant for FOX 2–4, FOX 2–5, FOX 2–6, and FOX 3–5.

##### 2.1.3.1 Sit1-Specific Uptake Assay

**Figure 4:**
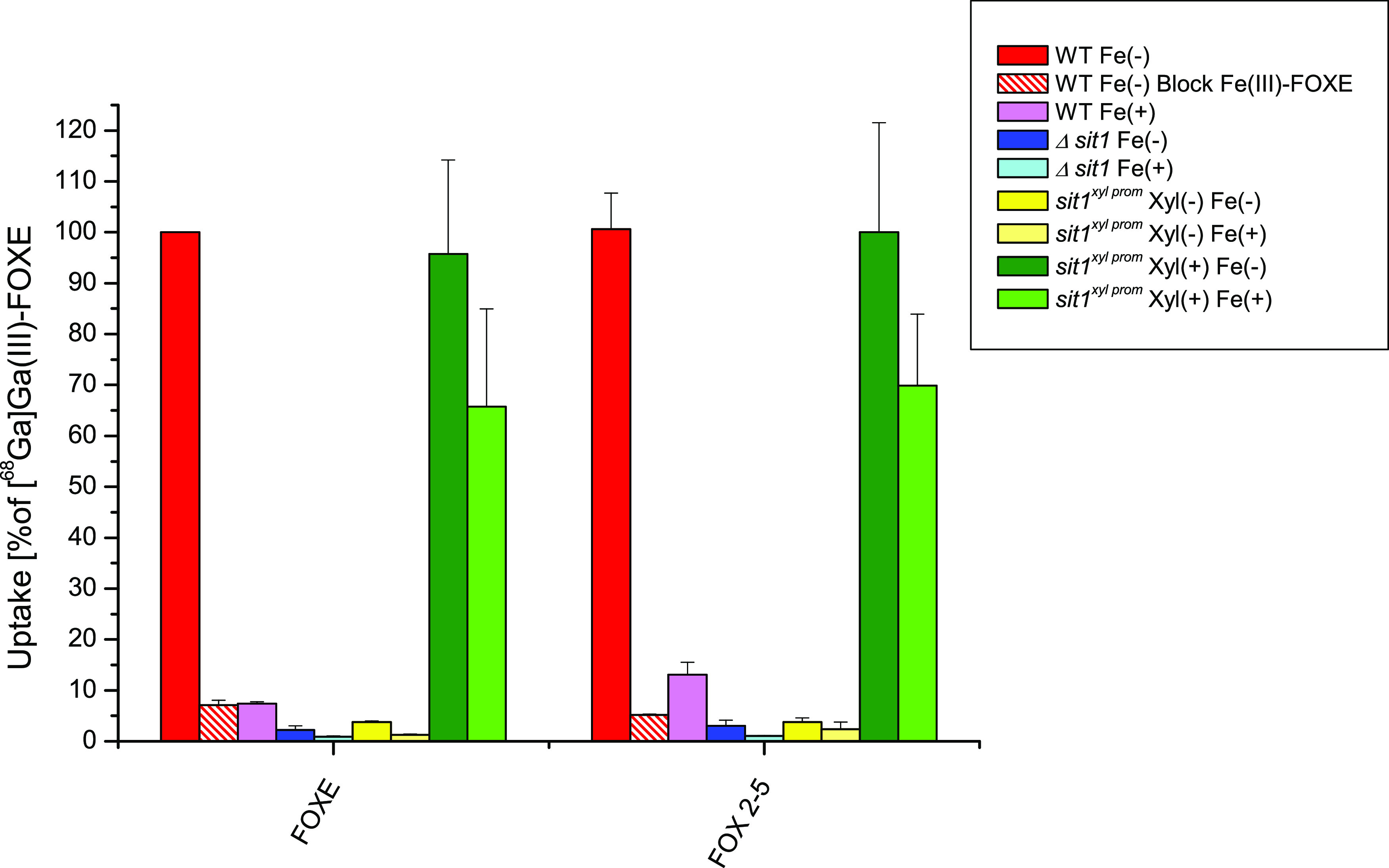
Uptake of [^68^Ga]Ga(III)-FOXE and [^68^Ga]Ga(III)-FOX 2–5 by *A. fumigatus* WT, Δ*sit1*, and *sit1*^*xyl prom*^. Uptake of [^68^Ga]Ga(III)-FOXE was normalized to WT. The results indicate relevant uptake only by the WT under iron deplete conditions (Fe(−)) and in the *sit1*^*xyl prom*^ mutant with xylose (Xyl(+)), which promotes artificial Sit1 upregulation, in contrast to the *sit1*^*xyl prom*^ mutant without xylose (Xyl(−)), a mutant lacking Sit1 and WT grown under iron-sufficient conditions (Fe(+)); (mean of four replicates from one experiment).

Growth promotion assays indicated that the uptake of all FOXE analogues depends on Sit1 (Figure 2). To investigate the specificity of FOX 2–5 for Sit1, short-term uptake of [^68^Ga]Ga(III)-FOX 2–5 was compared to that of [^68^Ga]Ga(III)-FOXE using three *A. fumigatus* strains, WT, Sit1-lacking Δ*sit1*, and *sit1*^*xyl prom*^, which expresses *sit1* under control of the *xylP* promoter allowing repression in the absence and induction in the presence of xylose largely independent of iron availability.^19,45^ Uptake was measured in iron-starved (Fe(−)) and iron-sufficient (Fe(+)) cultures as the expression of Sit1 is transcriptionally suppressed by iron in WT strains.^46^ As shown in Figure 4, [^68^Ga]Ga(III)-FOXE and [^68^Ga]Ga(III)-FOX 2–5 showed a largely identical uptake pattern: high uptake by mycelia of iron-starved WT and xylose-induced iron-starved as well as iron-replete *sit1*^*xyl prom*^. The slightly lower uptake by xylose-induced *sit1*^*xyl prom*^ mycelia under iron sufficiency compared to iron starvation is in agreement with the previously reported lower expression of xylose-induced *sit1* under iron sufficiency compared to iron starvation.^45^ In contrast, uptake of both siderophores was negligible by mycelia of iron-replete WT, FOXE-blocked WT, iron-starved as well as iron-replete Δ*sit1*, and xylose-noninduced *sit1*^*xyl prom*^ under both iron starvation and sufficiency.

### 2.2 *In Vivo* Characterization

#### 2.2.1 *Ex Vivo* Biodistribution of ^68^ Ga-Labeled FOXE Derivatives in Healthy Balb/c Mice

**Figure 5:**
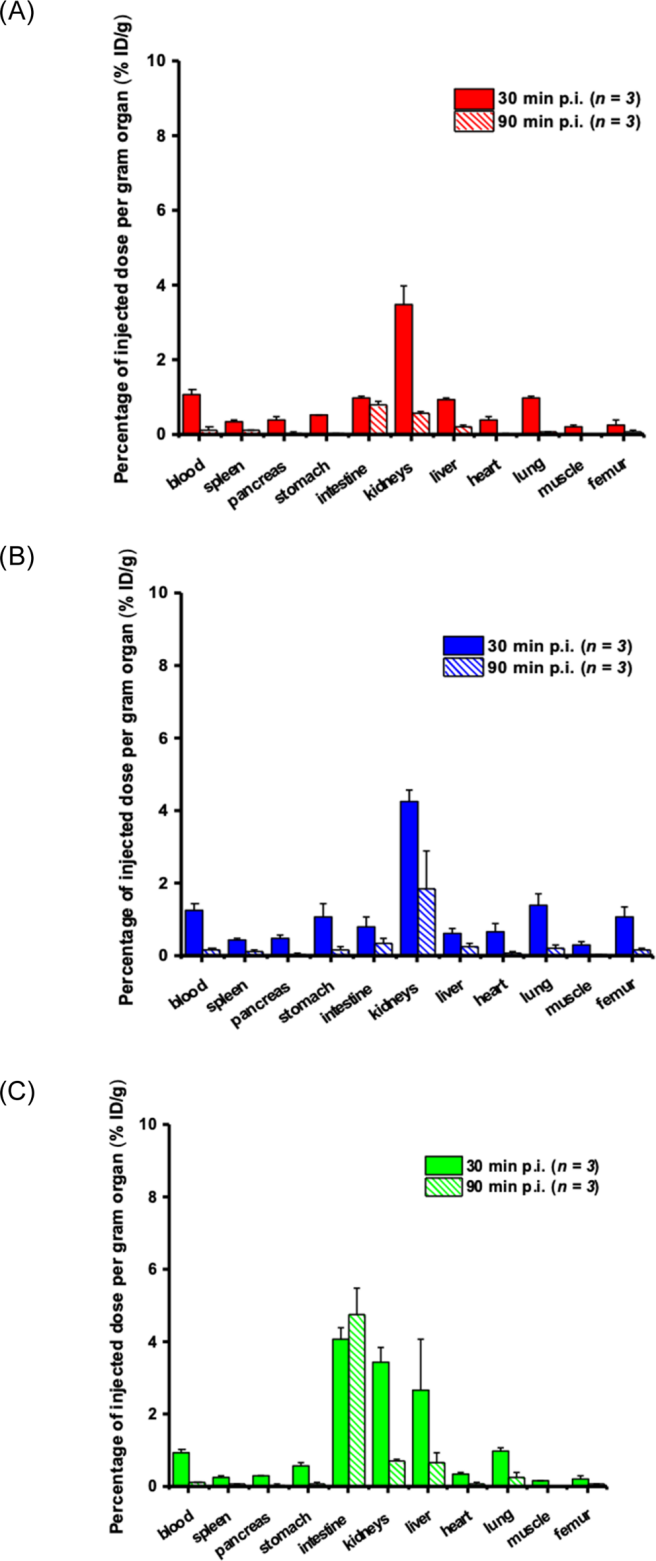
*Ex vivo* biodistribution data of (A) [^68^Ga]Ga-FOX 2–5, (B) [^68^Ga]Ga-FOX 2–4, and (C) [^68^Ga]Ga-FOX 2–6 in healthy Balb/c mice 30 and 90 min postinjection (*n* = 3 per time interval).

To investigate the pharmacokinetics of the FOXE analogues, *ex vivo* biodistribution studies were performed in which healthy Balb/c mice were injected with a radiolabeled compound and then, the level of radioactivity was measured in the blood and in the organs of interest (Figure 5).

[^68^Ga]Ga(III)-FOX 2–5 in healthy mice showed favorable pharmacokinetic properties promising for imaging applications, where significant retention was observed neither in the blood nor in any studied organ. The investigated compound was rapidly excreted by the kidneys, and only minimal radioactivity was excreted via the gastrointestinal tract with almost absent liver activity.

[^68^Ga]Ga(III)-FOX 2–4 displayed a very similar pharmacokinetic profile with slightly higher retention in some organs and slower excretion.

In contrast, [^68^Ga]Ga(III)-FOX 2–6 exhibited significantly different *in vivo* behavior, with higher levels of the radiotracer detected in the intestine and liver. The level of radioactivity in the intestine increased over time, whereas it decreased in all other organs.

#### 2.2.2 [^68^ Ga]Ga(III)-FOX 2–5 PET Imaging in the *A. fumigatus* Rat Infection Model

**Figure 6:**
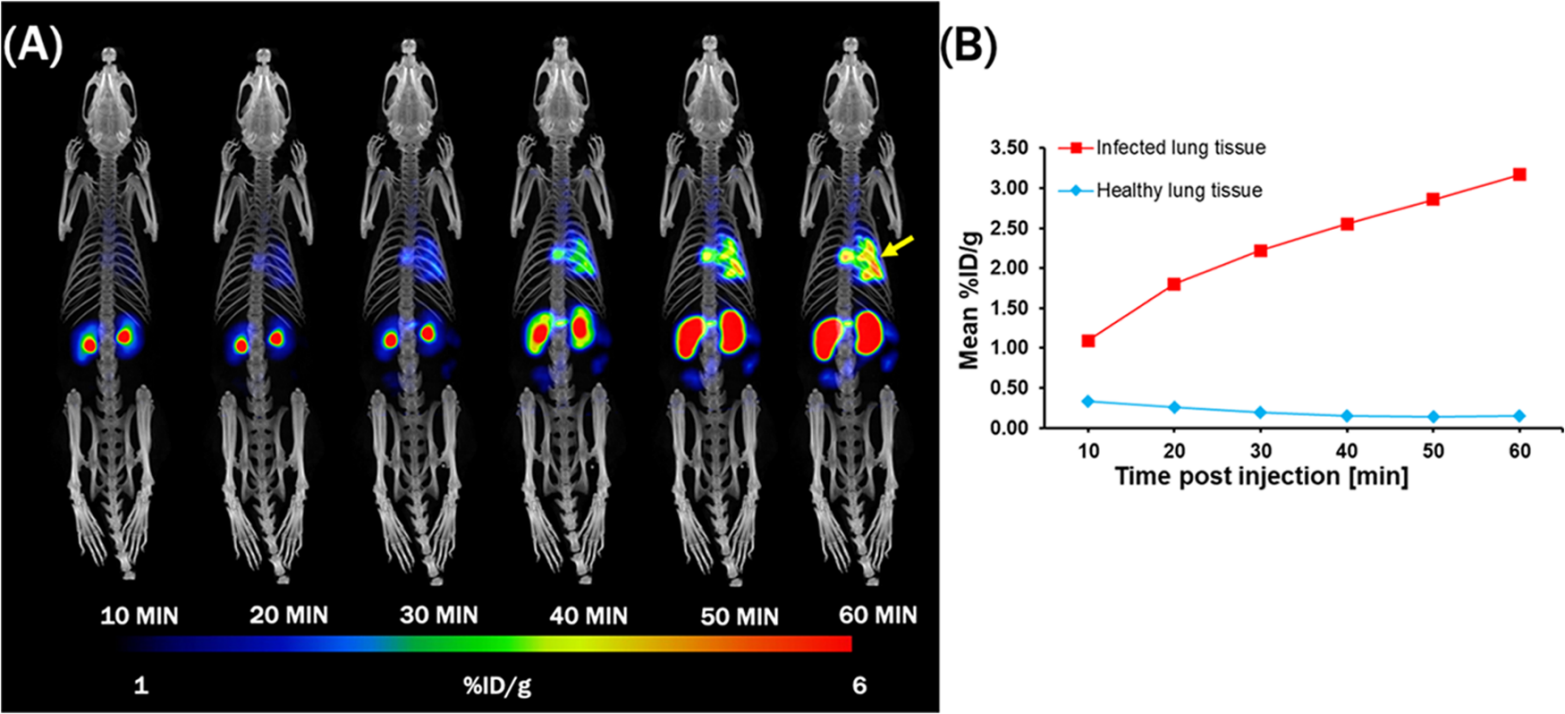
(A) Dynamic imaging (PET/CT MIP images) of [^68^Ga]Ga(III)-FOX 2–5 in a rat (*n* = 1) with *A. fumigatus* lung infection (48 h after infection) showing rapid uptake in the infected area and renal clearance of the unbound tracer up to 60 min postinjection. The yellow arrow indicates the site of infection. (B) Time–activity curves of [^68^Ga]Ga(III)-FOX 2–5 in healthy and infected rat lung tissue from (A).

Dynamic PET/CT imaging of [^68^Ga]Ga(III)-FOX 2–5 in a rat model of *A. fumigatus* lung infection showed rapid focal accumulation of [^68^Ga]Ga(III)-FOX 2–5 in specific areas of the lung (Figure 6). The images revealed a gradual increase in the radiotracer in infected lungs over time. The highest accumulation of the radiotracer with the best contrast was achieved at 60 min after injection. No detectable washout was observed in infected tissue over the whole imaging time. Radioactivity also accumulated rapidly in the kidneys, and only very minor radioactive signals were observed in the gastrointestinal tract, indicating predominant renal excretion of the tracer. These data are fully consistent with the results from *ex vivo* biodistribution studies.

**Figure 7:**
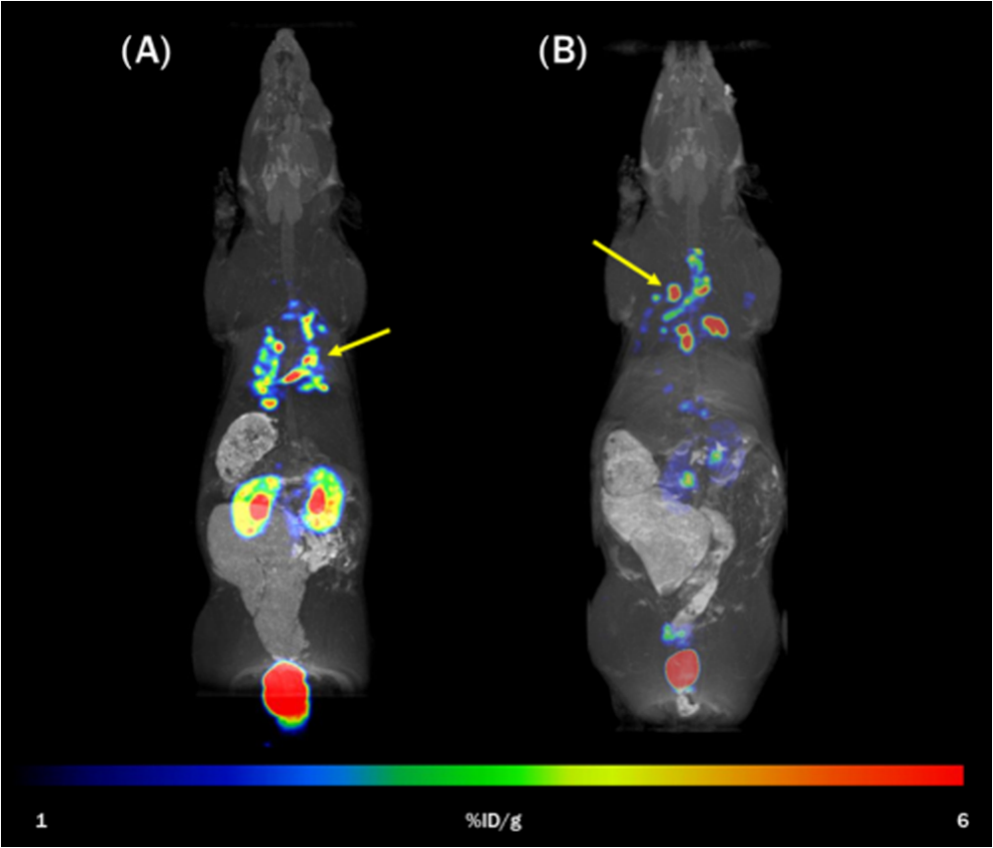
Static PET/MRI MIP images of [^68^Ga]Ga(III)-FOX 2–5 in a rat model of *A. fumigatus* lung infection. Images of different rats imaged (A) 48 h and (B) 72 h after infection and 45 min after injection of [^68^Ga]Ga(III)-FOX 2–5. Yellow arrows indicate the site of the infection.

Static PET/MRI maximum intensity projection (MIP) images of the rat respiratory *A. fumigatus* infection model showed significant focal accumulation of [^68^Ga]Ga(III)-FOX 2–5 in the infected lung and a major route of excretion through the urinary system (Figure 7). Very similar results were obtained for both rats imaged 48 h and 72 h after inoculation.

### 2.3 Species Specificity of FOXE Analogues

#### 2.3.1 Comparison of In Vitro Uptake of ^68^Ga-Labeled FOXE Derivatives in *A. fumigatus* and *Staphylococcus aureus* (*S. aureus*) Cultures

**Figure 8:**
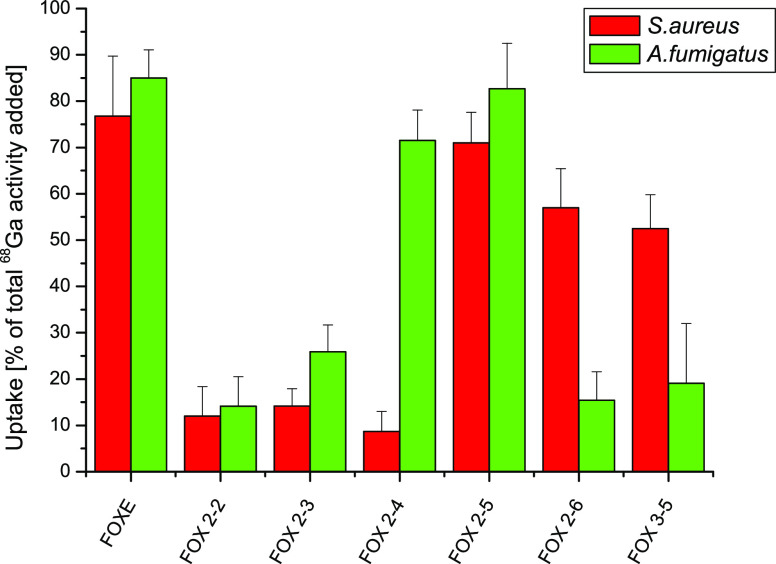
Comparison of ^68^Ga-labeled FOX derivatives uptake in *A. fumigatus* and *S. aureus* cultures. (mean of three independent experiments).

The results of the short-term active transport of FOXE derivatives by *A. fumigatus* and *S. aureus* in iron-deficient cultures showed an interesting pattern. By comparing data obtained previously for *S. aureus* uptake^42^ with those of *A. fumigatus* presented in this work, a species-specific behavior can be seen (Figure 8). While in the case of the native FOXE siderophore and FOX 2–5 analogue, high radioactivity levels were noted for both microorganisms, in the case of FOX 2–4, high radioactivity levels were seen only in *A. fumigatus* cultures (no specific signal was detected for *A. fumigatus* cultures incubated with the remaining FOX analogues). In contrary, in the case of FOX 2–6 and FOX 3–5, high radioactivity levels were seen in *S. aureus* cultures,^42^ while other compounds did not show significant biological activity.

The relative arrangement of hydroxamate and amide groups in FOX 2–6 and FOX 3–5 seems to be almost equally tolerated by *S. aureus*. In the case of FOX 2–5, the biological activity of the natural compound was retained, and this biomimetic derivative showed significant activity in both investigated microorganisms.

#### 2.3.2 Comparison of [^68^Ga]Ga(III)-FOX 2–4 vs [^68^Ga]Ga(III)-FOX 2–6 Activity in *A. fumigatus* and *S. aureus* Infection Models

**Figure 9:**
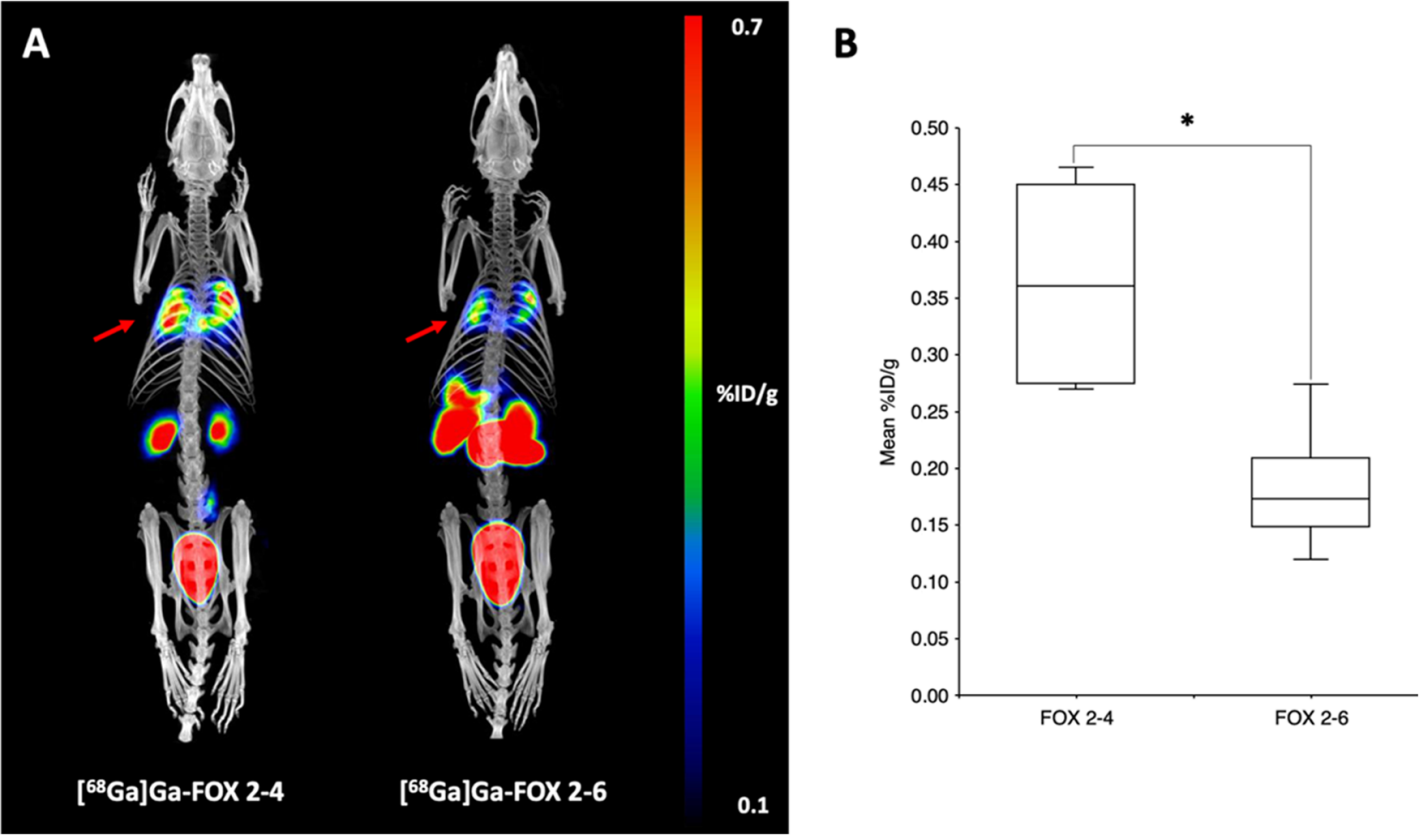
(A) Static PET/CT imaging data: MIP images of [^68^Ga]Ga-FOX 2–4 and [^68^Ga]Ga-FOX 2–6 in *A. fumigatus*-infected rats 45 min after injection. The red arrow indicates *A. fumigatus* infection. (B) Comparison of radioactive signal uptake in the lungs of *A. fumigatus*-infected rats (*n* = 4). Results are expressed as the mean of percentage of injected dose per gram of organ (%ID/g); *P < 0.05.

PET/CT imaging of *A. fumigatus*-infected animals displayed clear accumulation of [^68^Ga]Ga(III)-FOX 2–4 in infected lungs (Figure 9). Certain uptake in the infected lung region was observed also for [^68^Ga]Ga(III)-FOX 2–6 but with significantly lower intensity (0.36 ± 0.09 for [^68^Ga]Ga(III)-FOX 2–4 vs 0.19 ± 0.06 for [^68^Ga]Ga(III)-FOX 2–6; *P < 0.05). For [^68^Ga]Ga(III)-FOX 2–4, only visible noninfected organs were the kidneys and bladder, while in the case of [^68^Ga]Ga(III)-FOX 2–6, the contrast was significantly lower with an intense signal in the gastrointestinal and hepatobiliary system, which is in accordance with the previous results obtained from *ex vivo* biodistribution studies.

**Figure 10:**
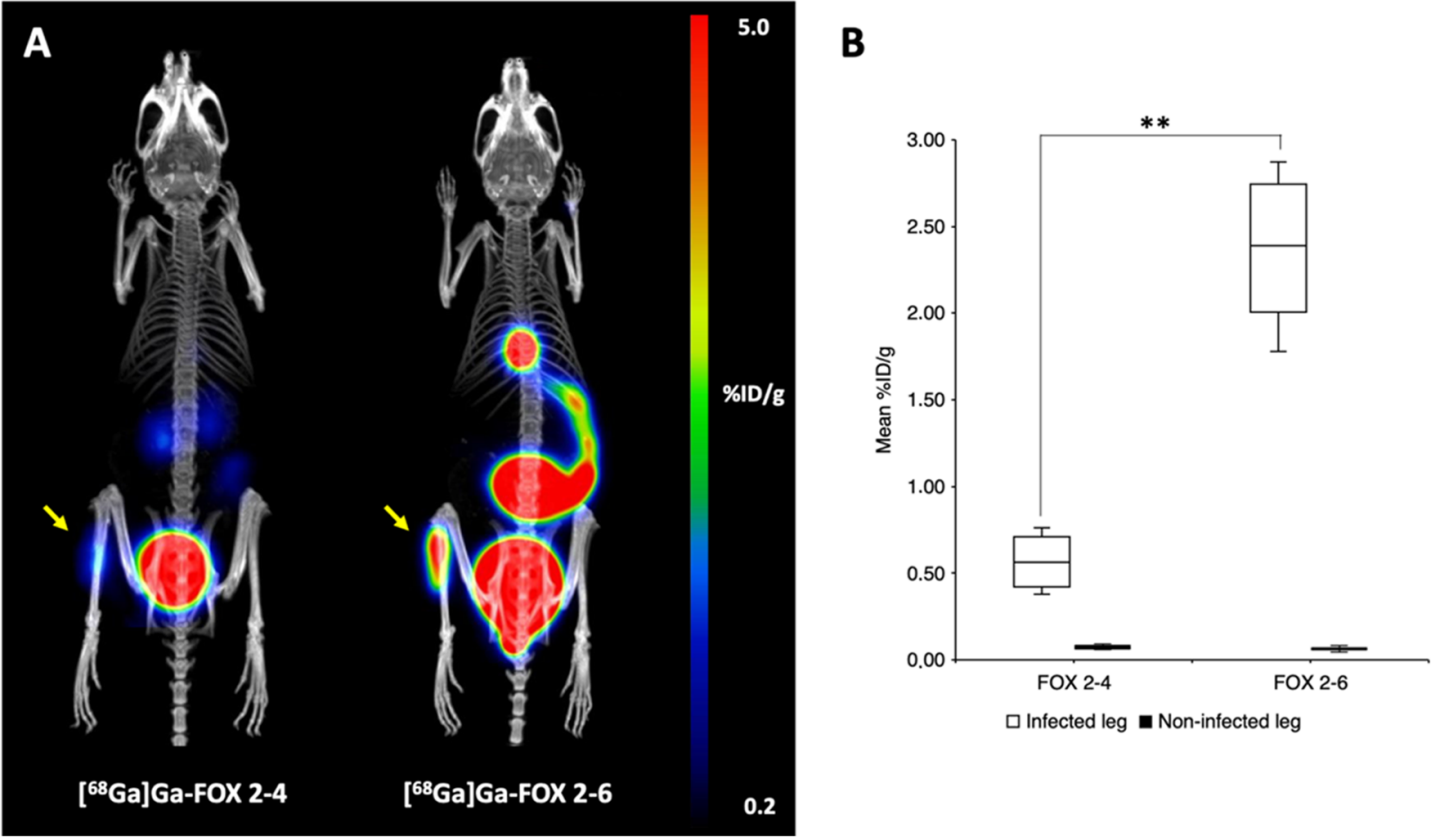
(A) Static PET/CT imaging data: MIP images of [^68^Ga]Ga(III)-FOX 2–4 and [^68^Ga]Ga(III)-FOX 2–6 in *S. aureus*-infected Balb/c mice 45 min after injection. Yellow arrows indicate *S. aureus* infection. (B) Comparison of radioactive signal uptake in the muscles of *S. aureus*-infected mice (*n* = 4). Results are expressed as the mean of percentage of injected dose per gram of organ (%ID/g); **P < 0.01.

PET/CT images of acute murine myositis displayed specific accumulation of [^68^Ga]Ga(III)-FOX 2–6 in *S. aureus* infection induced in the left hind limb of mice (Figure 10). Certain uptake in the infected limb was observed also for [^68^Ga]Ga(III)-FOX 2–4 but with significantly lower intensity (2.36 ± 0.44 for [^68^Ga]Ga(III)-FOX 2–6 vs 0.57 ± 0.16 for [^68^Ga]Ga(III)-FOX 2–4; **P < 0.01) comparable to the uptake in the gastrointestinal region. In the case of [^68^Ga]Ga(III)-FOX 2–6, a signal of high intensity was seen in the gastrointestinal tract with significant accumulation of the radiotracer in the gallbladder. The significantly different pharmacokinetics of the two examined compounds are in full accordance with *ex vivo* biodistribution studies.

## 3 Discussion

In our previous work, we have determined full solution chemistry of FOXE analogue complexes with Fe(III) and Ga(III) ions and their biological activity in vitro in *S. aureus* cultures.^42^ It is noteworthy that the radiolabeling process of FOX analogues with ^68^Ga(III) required no drastic conditions and was conducted at room temperature, at pH = 4.5, and the compounds were ready to use in no longer than 10 min. In this study, we have focused on the biological activity of these compounds in *A. fumigatus* and the effect of FOXE derivatization on species specificity.

In a first step, we investigated if Fe(III) complexes of the FOXE analogues are able to mimic native FOXE with regard to growth promotion of *A. fumigatus* employing the Δ*sidA*Δ*ftrA* mutant strain, which lacks endogenous siderophore production and reductive iron assimilation to avoid any interference with endogenous high-affinity iron uptake systems.^10,44^ The artificial siderophores were utilized with different efficacies following the order FOX 2–5 > FOX 2–6 > FOX 2–4 > FOX 3–5, while the uptake of FOX 2–2 and FOX 2–3 was negligible. Taken together, analogues with higher similarity to the natural counterpart (FOX 2–4, 2–5, 2–6, and 3–5) stimulated fungal colony growth in contrast to smaller analogues (FOX 2–2 and FOX 2–3). Growth promotion assays employing the Sit1-lacking Δ*sidA*Δ*ftrA*Δ*sit1* mutant demonstrated the exclusive uptake dependence of all FOXE analogues on Sit1 as shown previously for FOXE, FOB, and ferrioxamine G.^19^ This pattern indicates that the overall size of the mimicking molecule, modulated here by the spacer length and composition between the metal ion binding hydroxamate groups, is crucial for molecular recognition by Sit1. The retro-positioning of the hydroxamate groups was well tolerated by *A. fumigatus* Sit1, a behavior observed previously for retro-ferrioxamines^47^ and -ferrichromes^38−40^ and other retro-analogues of hydroxamate siderophores.^41^ Hampering of the biological activity of the analogues might be caused by different constraints, such as less effective iron chelation caused by either increased steric hindrance (in the case of smaller FOX analogues) or greater distance from the donor atoms (larger FOX analogues), suboptimal geometry and conformational freedom of the complex, or inappropriate position of amide groups for noncovalent interactions with the transporter. As demonstrated by pH-dependent solution thermodynamic studies,^42^ any change in FOXE size decreases the Fe(III) (and Ga(III)) ion binding strength with the stability constants of the forming complexes following the order FOXE > FOX 2–5 > FOX 2–4 > FOX 3–5 > FOX 2–3 > FOX 2–2. Nevertheless, the thermodynamic stability of complexes is exceptional and sufficient to prevent cross-chelation in the presence of other strong ligands, such as transferrin, present under biological conditions.^42^

*In vitro* studies of the radiolabeled FOXE derivative uptake confirmed that FOX 2–5 and FOX 2–4 can efficiently deliver radioactive gallium ions to the fungal cells. FOX 2–6 and FOX 3–5, which exhibited growth promotion activity, displayed significantly lower short-term uptake, while FOX 2–2 and FOX 2–3 lacked specific short-term uptake in agreement with the largely lacking growth promotion. Important to note is that the growth promotion assays provide information on the long-term activity of tested analogues, i.e., their ability to serve as an iron source over 48 h and their ability to promote the growth of microbial colonies acting as a source of iron. On the other hand, short-term uptake assays evaluate the uptake of a complex with a radiotracer within 1 h. In this regard, FOX 2–4 and FOX 2–5 were found to serve as efficient radiotracer carriers that mediate rapid cellular uptake of radioactive gallium ions in *A. fumigatus*. Short-term uptake assays employing *A. fumigatus* Sit1-lacking Δ*sit1* and *sit1*^*xyl prom*^, which allows conditional expression of *sit1*, confirmed the exclusive uptake dependence of all FOXE analogues by Sit1 already indicated by the growth promotion assays.

To translate *in vitro* studies into the conditions of a living complex organism and to assess the pharmacokinetics of FOXE analogues, healthy Balb/c mice were injected with radiolabeled FOXE analogues, and then, the radioactivity level was measured in organs of interest. In the case of FOX 2–4 and FOX 2–5, the results are very promising. Administered radiotracers were excreted from the living organism through the renal system, while other organs were essentially free of radioactive signals. This finding suggests that those complexes that do not interact with mammalian systems do not accumulate in any healthy tissue and that the radiotracer remains bound to the carrier molecule and is excreted efficiently from the organism. In contrast, data obtained for the FOX 2–6 analogue showed a different pattern with higher levels of radioactivity detected in the intestine and liver, suggesting an additional excretion route through the gastrointestinal tract with involvement of the hepatobiliary system. Taking into account our previous findings, this can be explained by the considerably more lipophilic character of [^68^Ga]Ga-FOX 2–6 (logD for FOX 2–4 = −3.02 ± 0.03, FOX 2–5 = −1.90 ± 0.03, FOX 2–6 = −0.58 ± 0.01).^42^ This is supported by the fact that the hydrophobic character of the FOX 2–6 analogue prevented solution chemistry studies of its complexes, as it was insoluble in water in the concentrations required for the implemented methods.^42^

Because of the best properties exhibited in the previous experiments, at this point, FOX 2–5 was chosen for further examination *in vivo*. The radiolabeled compound was assessed in a rodent infection model where aspergillosis was induced in the animal lungs.^35^ Dynamic PET/CT imaging allowed tracking of the pharmacokinetics of the radiotracer and revealed its clear focal accumulation in the infected tissue, while no significant signal was detected in other organs. Radioactivity rapidly accumulated in the kidneys, suggesting efficient excretion through the renal system. However, no detectable washout was observed in the infected tissue over the whole imaging time, indicating that the accumulation of the radiotracer in the fungal cells is highly efficient. Additionally, static PET/MRI imaging in *A. fumigatus*-infected rats further supported these results. Taken together, FOX 2–5 retained the biological activity under the conditions of a living organism and was selectively accumulated by *A. fumigatus* cells in the infected lung tissue. This finding is of high importance, as microbial-selective probes are scarce.

The comparison of *in vitro* uptake of ^68^Ga-labeled FOXE derivatives in *A. fumigatus* and *S. aureus*(42) cultures clearly revealed species-specific assimilation of analogues differing from the natural FOXE in size and the retro-arrangement of the hydroxamate binding groups relative to the amide groups. The shortening of the spacers between the hydroxamate groups by one methylene unit, −CH_2_–, allowed FOX 2–4 to be selectively recognized and assimilated by *A. fumigatus*. Analogous elongation of the spacers in FOX 2–6 and 3–5 brought a higher specificity for *S. aureus*.^42^ The relative arrangement of hydroxamate and amide groups in FOX 2–6 and FOX 3–5 seems to be equally tolerated by *S. aureus*. This finding is very promising as species specificity is not exhibited by the native FOXE or the most alike analogue FOX 2–5, which were both highly active in both microorganisms. To validate the hypothesis of species-specific transport of biomimetic FOXE analogues and translate the *in vitro* findings into *in vivo* conditions, static PET/CT assays were repeated with the respective analogues in the animal models of pulmonary aspergillosis and compared with images obtained from a mouse model with bacterial muscle infection. The results were in full accordance with the pattern observed *in vitro*. FOX 2–4 exhibited significantly higher radioactivity accumulation in infected lung tissue as compared to FOX 2–6 in the aspergillosis model, whereas in the PET scans of acute murine myositis in mice, radiolabeled FOX 2–4 displayed 4 times lower accumulation in the limb inoculated with *S. aureus* compared to radiolabeled FOX 2–6. In both studies, significant radioactivity levels were detected in the gastrointestinal region for the FOX 2–6 analogue, confirming the involvement of the hepatobiliary tract in the excretion for this analogue, in contrast to FOX 2–4, which showed only renal excretion very similar to FOX 2–5.

Overall, the modification of the size of the FOXE ring and the arrangement of the hydroxamate binding groups in relation to the amide groups allowed identification of the compounds promoting *in vitro* and *in vivo* species specificity for the fungal species *A. fumigatus* versus the Gram-positive bacterial species *S. aureus*.

For both pathogens, FOXE is a xenosiderophore, but their cellular uptake systems differ, which should explain the observed species specificity of the FOXE analogues. We have previously shown that uptake of ferrioxamines, such as FOXE and FOB, in *A. fumigatus* depends on the major facilitator transporter-type Sit1^19,45^ and demonstrated the same for the FOXE analogues here. In contrast, *S. aureus* employs the extracellular surface lipoproteins FhuD1 and FhuD2 along with the ABC transporter FhuC to facilitate the use of hydroxamate xenosiderophores such as FOB for iron acquisition.^48−51^ Sit1 and FhuD1/2 belong to different protein families with highly diverse structures: Sit1 is a major facilitator consisting of 14 trans-membrane α-helices,^19^ while FhuD1/2 has a bilobate bean-like structure typical of the class III solute binding proteins (SBP), composed of two globular domains consisting of β-strands and α-helices with connection of the domains by a long α-helix.^52^ Among the hydroxamate siderophores tested, FhuD2 seems to have higher affinity and substrate flexibility than FhuD1.^27,50,52,53^

## 4 Conclusions

This study delivered a new series of compounds with very promising properties as selective and efficient agents for the detection of invasive aspergillosis. The designed biomimetic analogues of a natural FOXE siderophore retain the biological activity of their native counterpart. The most structurally similar analogue, FOX 2–5 delivered radioactivity to the fungal cells exclusively via Sit1, mimicking the function of natural ferrioxamines. This capability was successfully translated to an animal infection model, where the proposed analogues exhibited high metabolic stability and favorable pharmacokinetics with rapid renal excretion in aspergillosis-induced animals. High specific uptake in *A. fumigatus* was confirmed by PET/CT and PET/MRI scans, which allowed for a quick and precise localization of the infection. Interestingly, this study also revealed species specificity in the biological activity of some analogues. While the most alike analogue FOX 2–5 to native siderophore FOXE presented the same broad spectrum of activity, other analogues showed a different behavior. FOX 2–4 was particularly active in *A. fumigatus* and only slightly active in *S. aureusin vitro*. On the contrary, for FOX 2–6 and FOX 3–5, high biological activity was seen in *S. aureus* but not in *A. fumigatus*. This behavior was confirmed in imaging studies in the respective microbial infection models. Further studies will follow to investigate the observed species specificity and strain differences in uptake selectivity and growth promotion. Similar research involving FOX analogues is now continued by the authors with regard to *P. aeruginosa* infection models.

This finding is of high importance, as it demonstrates the possibility of tuning the biological properties of siderophores. Moreover, this work delivers a promising series of biologically active compounds that can be radiolabeled under mild conditions. As radioactive probe carriers that do not require drastic conditions for preparation are scarce, the studied series of compounds present potential for future research, not only as sole carriers but also in conjugates with functional entities.

Along with application studies, this work delivers valuable data about biomimetic chemistry, design of tailor-made screening compounds for selective targeting of pathogenic microorganisms, and structural requirements of siderophore recognition by cognate transporters. It also encourages further studies on molecular details of the recognition and transport of biomimetic analogues of FOXE in other pathogens.

## 5 Experimental Section

### 5.1 Chemicals

All commercially available chemicals were obtained as analytical grade and used without further purification. FOXE (nocardamine) was obtained commercially from Pol-Aura (Zabrze, Poland). The studied FOX compounds were synthesized commercially by TriMen Chemicals (Lod, Poland) and used as received. The 4-step synthetic strategy was described in detail, together with purity check and structure determination, in our previous work.^42^ The certificates of analysis of FOXE and FOX compounds are shown in the Supporting Information supplemented to this work (Characterization of Ligands). [^68^Ga]GaCl_3_ was obtained from a ^68^Ge/^68^Ga generator (Model IGG100, Eckert & Ziegler Isotope Products, Berlin, Germany) by fractionated elution, using 0.1 M HCl solution (Rotem Industries Ltd., Beer-Sheva, Israel).

### 5.2 Fungal Strains and Growth Conditions

The *A. fumigatus* strains used included AfS77 (termed WT here), Δsit1 (lacking Sit1), *sit1*^*xyl prom*^ (expressing *sit1* under control of the *xylP* promoter, which allows repression in the absence and induction in the presence of xylose supplementation), Δ*sidA*Δ*ftrA* (lacking siderophore biosynthesis and reductive iron assimilation), and Δ*sidA*Δ*ftrA*Δ*sit1* (lacking siderophore biosynthesis, reductive iron assimilation and Sit1). All strains have been previously described.^19,45^ Fungal strains were cultured at 37 °C in *Aspergillus* minimal medium (AMM)^54^ with 1% (w/v) glucose as the carbon source and 20 mM glutamine as the nitrogen source, using iron-free trace elements to induce iron limiting for the transcriptional activation of siderophore uptake.^46^ Iron-sufficient media contained FeSO_4_ in a final concentration of 30 μM. For iron-limiting conditions, the supplementation of iron was omitted.

### 5.3 Preparation of Complexes

The complexation of Fe(III) and Ga(III) requires mild conditions and takes place at room temperature, while the reaction is rapid. For the purpose of current studies, the complexes were obtained before use by mixing appropriate amounts of ligand and metal ions (vide infra). Full-solution thermodynamic studies and detailed physicochemical characterization of Fe(III) and Ga(III) FOXE complexes were described in our previous work, where coordination characteristics, thermodynamic stability, and complex formation equilibria of FOXE analogues with Fe(III) and Ga(III) ions were thoroughly investigated.^42^ For the summary of information on complex characteristics (ESI-MS, thermodynamic stability, and UV–vis characteristics), please see the Supporting Information supplemented to this work (Tables S1and S2).

#### 5.3.1 Ferric Complexes

Iron-containing Fe(III)-FOXE and Fe(III)-FOX were obtained by the addition of a 10-fold molar excess of FeCl_3_ in aqueous solution to FOXE or FOX with subsequent purification by preparative HPLC.

#### 5.3.2 Radiolabeling

10 μg of FOXE derivatives was mixed with 200 μL of [^68^Ga]GaCl_3_ (approximately 24 MBq) in 0.1 M HCl. The pH of the solution was adjusted to 4.5 by adding 40 μL of 1.1 M sodium acetate. Radiolabeling was carried out at room temperature with an incubation time of 10 min. Radiochemical yields of labeled ^68^Ga-FOXE derivatives were determined using RP-HPLC and/or radio-instant thin-layer chromatography and, if necessary, purified by solid-phase extraction as described previously (Table S3).^42^

### 5.4 *In Vitro* Characterization

#### 5.4.1 Short-Term Uptake Assay in *A. fumigatus*

Uptake assays were performed in both iron-deficient and iron-sufficient *A. fumigatus* cultures as described previously.^55^ The uptake of each [^68^Ga]Ga(III)-FOXE derivative was expressed as the ratio to [^68^Ga]Ga(III)-FOXE uptake from the same experiment in order to normalize uptake variations between different fungal cultures. The mean values of three different assays were used to quantitatively compare the difference in uptake between the compounds.

#### 5.4.2 Sit1 Specificity Assay

In order to analyze the role of the SIT1 importer for uptake of [^68^Ga]Ga(III)-FOXE derivatives, uptake assays were conducted in AfS77 Δsit1 strain as well as AfS77 sit1xylP strain, using both xylose-sufficient and xylose-deficient cultures.^45^ Assays were executed as prescribed above.

#### 5.4.3 Siderophore Utilization Assay of *A. fumigatus* via Growth Promotion

To investigate the ability of *A. fumigatus* to take up and utilize Fe(III)-FOXE derivatives as the iron source, a growth assay with *A. fumigatus* mutant strains *ΔsidA/ΔftrA and ΔsidA/ΔftrA/*Δ*sit1* was conducted. In a 24-well plate, aliquots of 10^3^ fungal conidia were point-inoculated on 1 mL of AMM containing 0.0, 0.1, 1, 10, or 50 μM Fe(III)-siderophore and incubated at 37 °C for 48 h.

### 5.5 *In Vivo* Characterization

#### 5.5.1 Animal Experiments

Animal experiments were performed in female 8–10-week-old Balb/c mice and female 2–3-month-old Lewis rats (Envigo, Horst, The Netherlands). The animals were acclimatized to laboratory conditions for 1 week prior to experimental use and housed under standard laboratory conditions on sawdust in individually ventilated cages with free access to animal chow and water. All experiments were conducted in accordance with regulations and guidelines of the Czech Animal Protection Act (No. 246/1992), with the approval of the Czech Ministry of Education, Youth, and Sports (MSMT-9847/2019–5 and MSMT-24421/2021–4), and the institutional Animal Welfare Committee of the Faculty of Medicine and Dentistry of Palacky University in Olomouc. During the experiments, the general health and body weight of the animals were monitored. The number of animals was reduced as much as possible (generally *n* = 3 per group and time point) for all *in vivo* experiments; small animal imaging was carried out under 2% isoflurane anesthesia (FORANE, Abbott Laboratories, Abbott Park, IL) to minimize animal suffering and to prevent animal motion.

#### 5.5.2 Animal Infection Models

A previously described rat model of *A. fumigatus* lung infection^56^ and a mouse model of *S. aureus* myositis were used for the *in vivo* infection experiments. Infection in the lung of immunodeficient rats was established by intratracheal inoculation of 100 μL of *A. fumigatus* spores (10^9^ CFU/ml *A. fumigatus* ATCC46645) using the TELE PACK VET X LED system equipped with a rigid endoscope (Karl Storz GmbH & Co. KG, Tuttlingen, Germany). Depending on the development of the infection, experimental animals underwent PET/CT or PET/MRI imaging, typically 2–3 days after inoculation.

Murine acute myositis model was established by intramuscular (i.m.) injection of bacteria. Immunodeficient mice were inoculated with 5 × 10^7^ CFUs of *S. aureus* in the left hind leg. The microbial infection was allowed to develop for 5 h, and infected animals were subsequently r.o. injected with [^68^Ga]Ga(III)-FOX 2–4 or [^68^Ga]Ga(III)-FOX 2–6 and imaged by means of PET/CT.

#### 5.5.3 Biodistribution Studies

To evaluate pharmacokinetics and biodistribution in healthy animals, a group of three Balb/c mice per time point were retro-orbitally (r.o.) injected with [^68^Ga]Ga(III)-FOX 2–4, [^68^Ga]Ga(III)-FOX 2–5, or [^68^Ga]Ga(III)-FOX 2–6 (1–2 MBq/mouse, approximately ∼0.5–1 μg of FOX derivative). Animals were sacrificed by cervical dislocation at 30 and 90 min postinjection (p.i.). Organs and tissues of interest (blood, spleen, pancreas, stomach, intestines, kidneys, liver, heart, lung, muscle, and bone) were collected, weighed, and measured in a γ counter. Results were expressed as a percentage of injected dose per gram of organ (%ID/g).

#### 5.5.4 Animal Imaging

Animal imaging was done as described previously.^55,57^ Briefly, anesthetized *A. fumigatus* infected rats or *S. aureus*-infected mice were r.o. injected with [^68^Ga]Ga(III)-FOX 2–5 (∼10 MBq/rat, approximately ∼2 μg of FOX 2–5) or [^68^Ga]Ga(III)-FOX 2–4 or [^68^Ga]Ga(III)-FOX 2–6 (∼10 MBq/rat, approximately ∼4 μg of FOX derivative and ∼8–6 MBq/mouse, approximately ∼4 μg of FOX derivative) and placed in the prone position in the respective imaging systems. Dynamic μPET/CT imaging was performed on a Mediso NanoScan PET/CT imaging system for small animals (Mediso Medical Imaging Systems, Budapest, Hungary). Single FOV (98.55 mm) PET scan was acquired at ∼5 to 60 min p.i., followed by whole-body helical CT (50 kVp/908 μA, 720 projections). μPET/3T MRI imaging was performed at 45 min p.i. using the Mediso NanoScan PET/MRI 3T small animal imaging system (Mediso Medical Imaging Systems, Budapest, Hungary). Static PET acquisition was performed for 20 min with double FOV PET scans (2 mm × 98.5 mm; 2 mm × 10 min), followed by a coronal T1-weighted 3D gradient echo scan (slice thickness = 0.6 mm; TR = 15 ms; TE = 3.9 ms; NEX = 2; flip angle = 20°). Static μPET/CT imaging was performed at 45 min p.i. using the Mediso NanoScan PET/CT system mentioned above. Single FOV PET scan (98.55 mm) followed by whole-body helical CT was used for dynamic imaging. Image reconstruction for data from both scanners was performed using Mediso Tera-Tomo iterative 3D PET reconstruction (Mediso Medical Imaging Systems, Budapest, Hungary). Image visualization, processing, and quantification were performed with Mediso InterView FUSION software (Mediso Medical Imaging Systems, Budapest, Hungary). All images were normalized to the injected activity and animal weight.

#### 5.5.5 Statistical and Data Analysis

Statistical analyses of animal experiments were performed using Microsoft Office 365 Excel (Microsoft Corporation, Redmond, WA). All bar graphs presented include error bars representing the standard deviation. Data from imaging studies were analyzed using an unpaired two-tailed Student’s *t* test. Box and whisker plots show medians and interquartile ranges, the horizontal line in each box indicates the median, and the whiskers indicate the lowest and highest values that are not classified as outliers.

## Supporting Information Available

Characterization of ligands, certificates of analysis, characterization of complexes, coordination properties of the studied ligands toward Fe(III) and Ga(III), intensity maxima of the major complexes and adduct ions observed by ESI-MS, overall stability constants, and radiochemical purity of [^68^Ga]GaFOX analogues as determined by SPE, HPLC, and TLC (PDF)
Molecular formula strings, SMILES (CSV)

## Abbreviations Used

CT: computed tomography
FOB: ferrioxamine B
FOXE, DFO E: ferrioxamine E, nocardamine, CAS Number: 26605–16–3
FOX: ferrioxamine E analogues investigated in this study
MIP: maximal intensity projection
MRI: magnetic resonance imaging
PET: positron emission tomography
RP-HPLC: reversed-phase high-performance liquid chromatography
SEP: solid-phase extraction
