# Supplementary material for: Biomimetic Analogues of the Desferrioxamine E Siderophore for PET Imaging of Invasive Aspergillosis: Targeting Properties and Species Specificity
Source: J Med Chem. 2024 Jun 22;67(14):12143–54. doi: 10.1021/acs.jmedchem.4c00887 (PMC11284789; doi:10.1021/acs.jmedchem.4c00887)
Supplement: Supplementary file 1 — jm4c00887_si_001.pdf [file jm4c00887_si_001.pdf]

Biomimetic Analogues of Desferrioxamine E Siderophore for PET Imaging of Invasive Aspergillosis: Targeting Properties and Species Specificity

Andrzej Mular[a], Isabella Hubmann[b], Milos Petrik[c], Katerina Bendova[c], Barbora Neuzilova[c], Mario Aguiar[b,d], Patricia Caballero[d], Abraham Shanzer[e], Henryk Kozłowski[a,f], Hubertus Haas[d], Clemens Decristoforo[b]*, and Elzbieta Gumienna-Kontecka[a]*

[a] Faculty of Chemistry, University of Wrocław, 50-383 Wrocław, Poland
email: elzbieta.gumienna-kontecka@uwr.edu.pl
[b] Department of Nuclear Medicine, Medical University Innsbruck, A-6020 Innsbruck, Austria
[c] Institute of Molecular and Translational Medicine, Faculty of Medicine and Dentistry and Czech Advanced Technology and Research Institute, Palacky University, 77900 Olomouc, Czech Republic
[d] Institute of Molecular Biology, Biocenter, Medical University Innsbruck, A-6020 Innsbruck, Austria
[e] Department of Organic Chemistry, The Weizmann Institute of Science, Rehovot 7610001, Israel
[f] Public Higher Medical Professional School in Opole, Katowicka 68, 45-060 Opole, Poland

Supporting Information

TABLE OF CONTENTS

1. Characterization of ligands……………………….………..…………………………S2

1.1. Certificates of analysis

2. Characterization of complexes……………………….…………..…………………… S9

2.1 Coordination properties of studied ligands towards Fe(III) and Ga(III)………………..…S9

Table S1. Intensity maxima of the major complexes and adduct ions observed by ESI-MS

Table S2. Overall stability constants (logβ_{ML} and pFe(III) and pGa(III) values)

2.2 Radiolabeling…………………….……………………………………………..…….S11

Table S3. Radiochemical Purity (RCP) of [^{68}Ga]GaFOX analogs as determined by SPE, HPLC and TLC

1. Characterization of ligands

FOXE (Nocardamine) was obtained commercially from Pol-Aura (Zabrze, Poland). Studied FOX compounds were synthesized commercially by TriMen Chemicals (Łodź, Poland) and used as received. The 4-step synthetic strategy was described in details, together with purity check and structure determination, in our previous work. [1] The certificates of analysis are shown in the following section.

1.1 Certificates of analysis

Name: **FOXE, Nocardamine**
Catalogue number: PS-034846-A
CAS Number: 26605-16-3
Mol. weight: 600.70 g/mol
Form: white powder, soluble in DMSO, methanol, ethanol or water
Keep at -20°C
Purity (HPLC), min. 95%

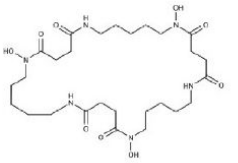

Name: **FOX 2-2**
Catalogue number: 0005117
Lot: JK-889-2
Mol. weight: 474.47 g/mol
Form: freeze-dried
Keep in the fridge
Purity: > 95% (by LCMS/NMR)

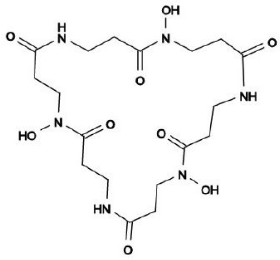

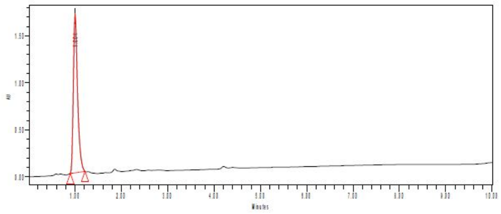

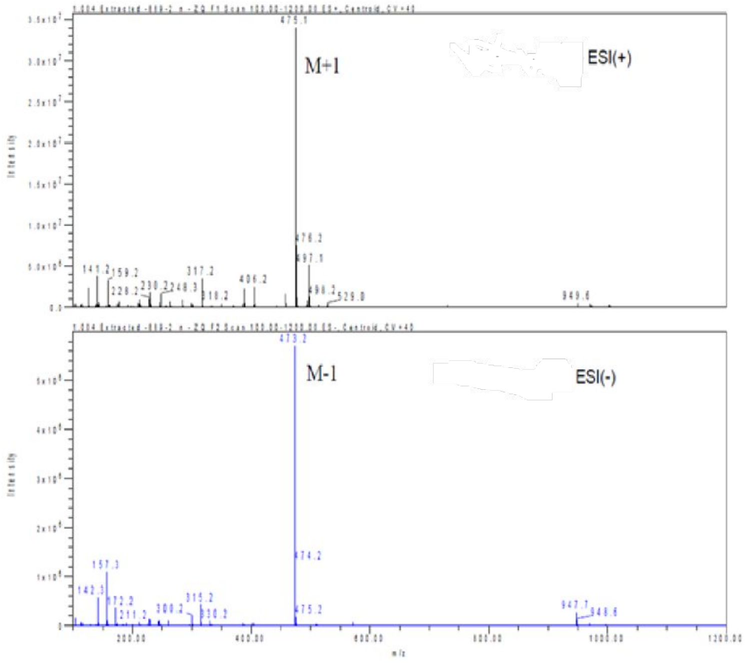

FOX 2-2: 98.4% (0-60% ACN)

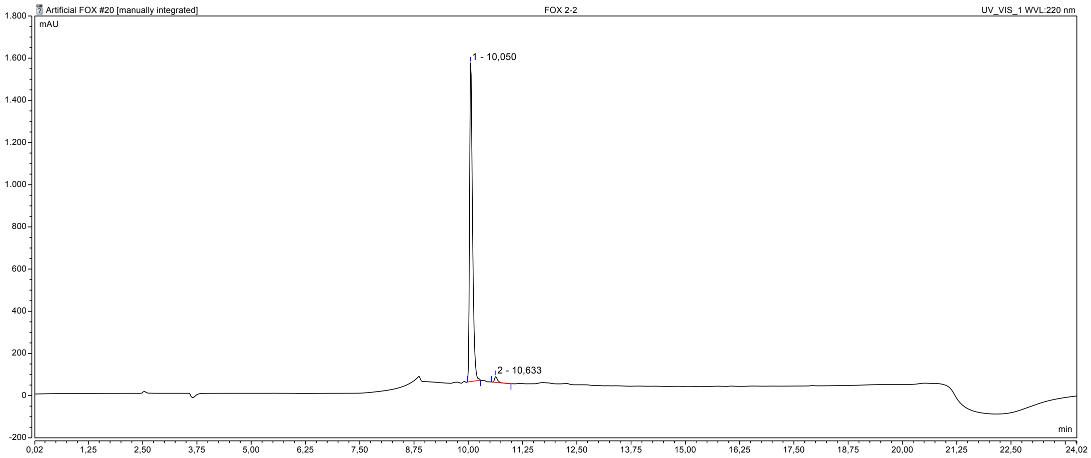

Name: **FOX 2-3**
Catalogue number: 0005118
Lot: JK-928
Mol. weight: 516.54 g/mol
Form: freeze-dried
Keep in the fridge
Purity: > 95% (by LCMS/NMR)

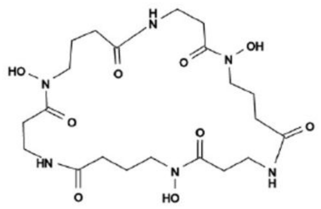

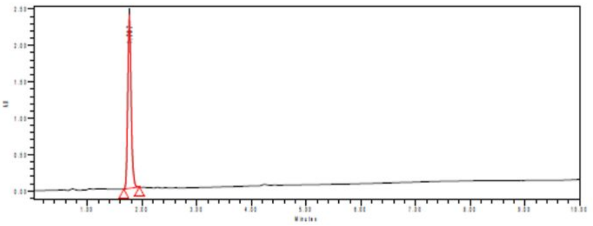

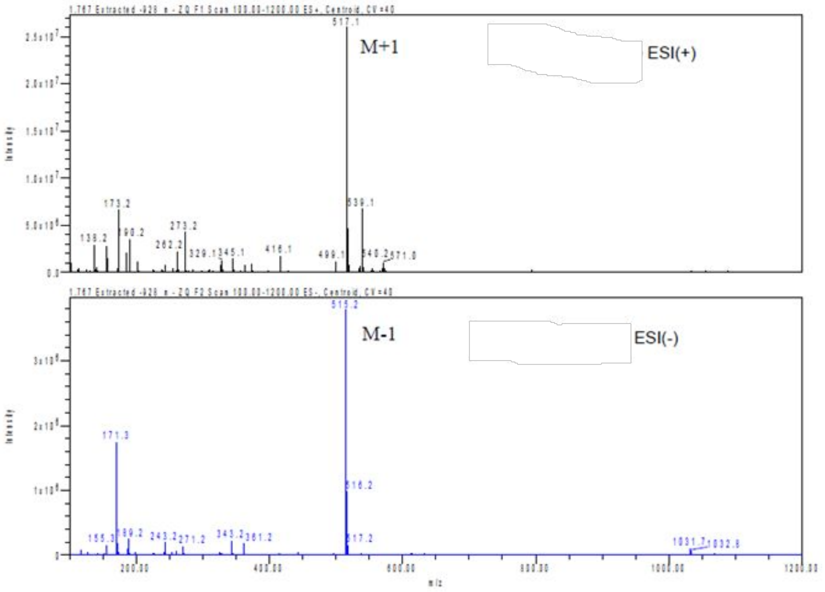

FOX 2-3: 95.4%(10-60% ACN)

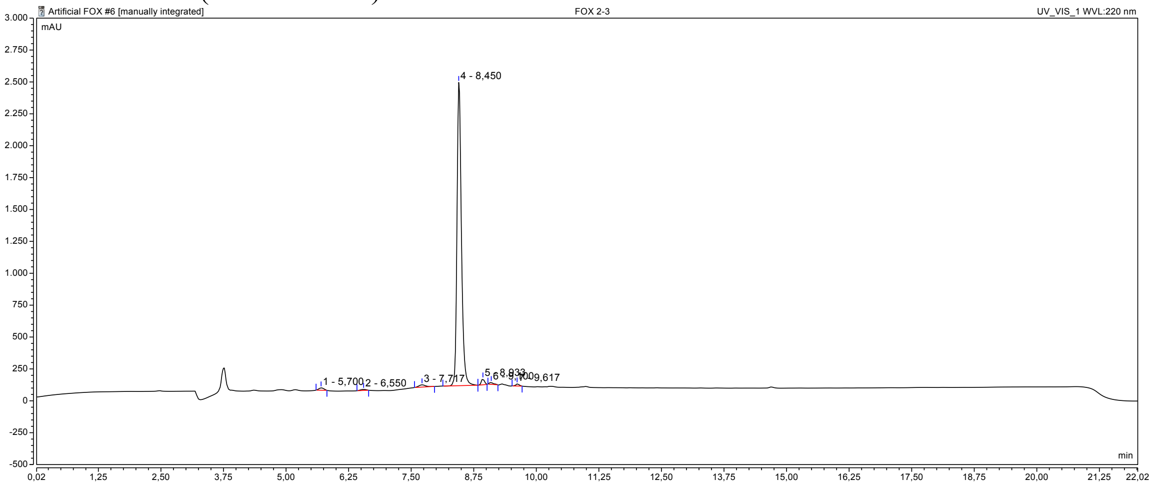

Name: **FOX 2-4**
Catalogue number: 0005119
Lot: JK-928
Mol. weight: 558.6 g/mol
Form: freeze-dried
Keep in the fridge
Purity: > 95% (by LCMS/NMR)

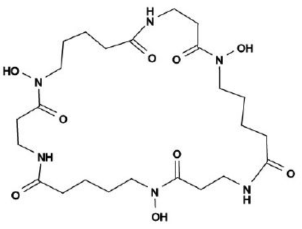

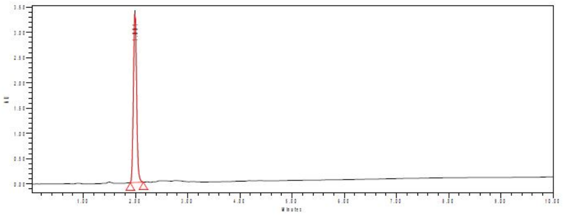

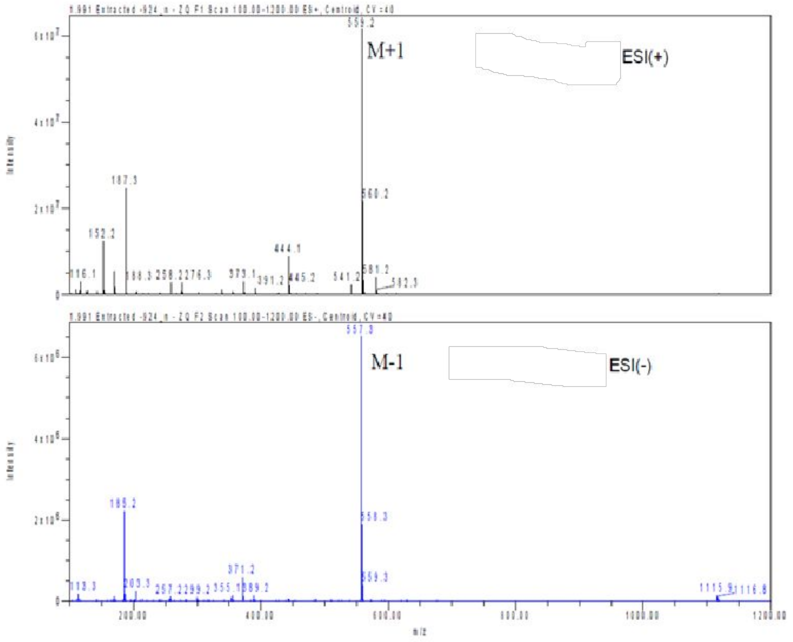

FOX 2-4: 97.8%(10-60% ACN)

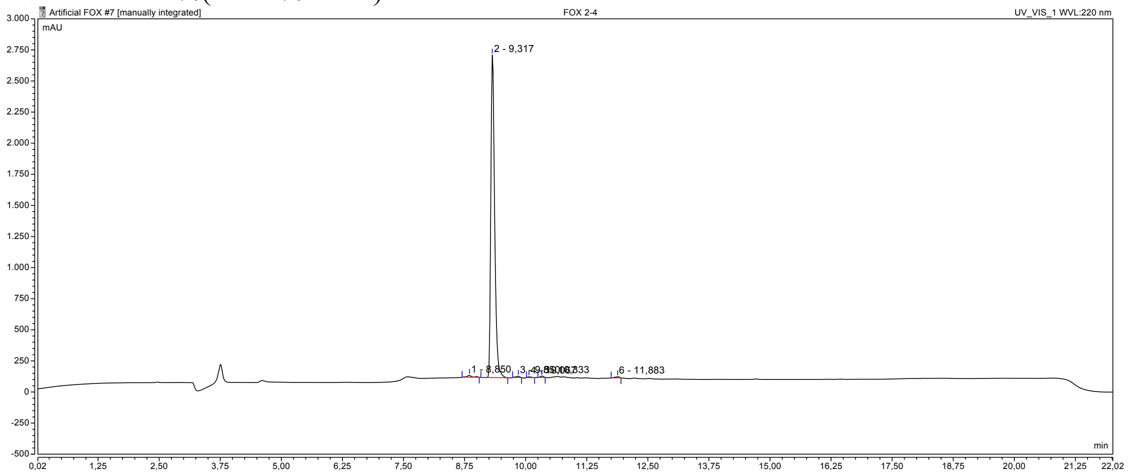

Name: **FOX 2-5**
Catalogue number: 0005120
Lot: JK-922
Mol. weight: 600.7 g/mol
Form: freeze-dried
Keep in the fridge
Purity: > 95% (by LCMS/NMR)

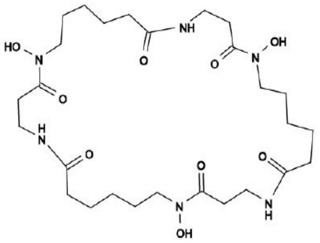

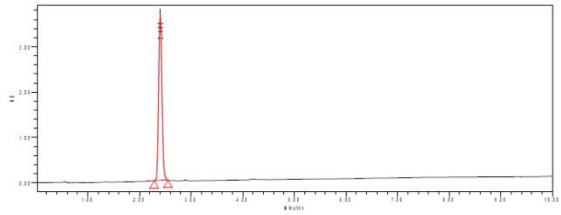

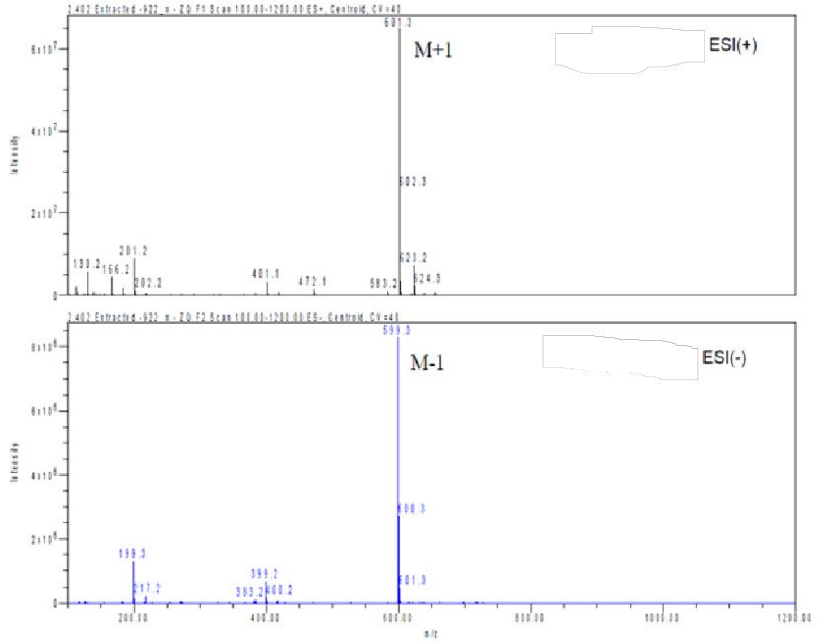

FOX 2-5: 96.6%(10-60% ACN)

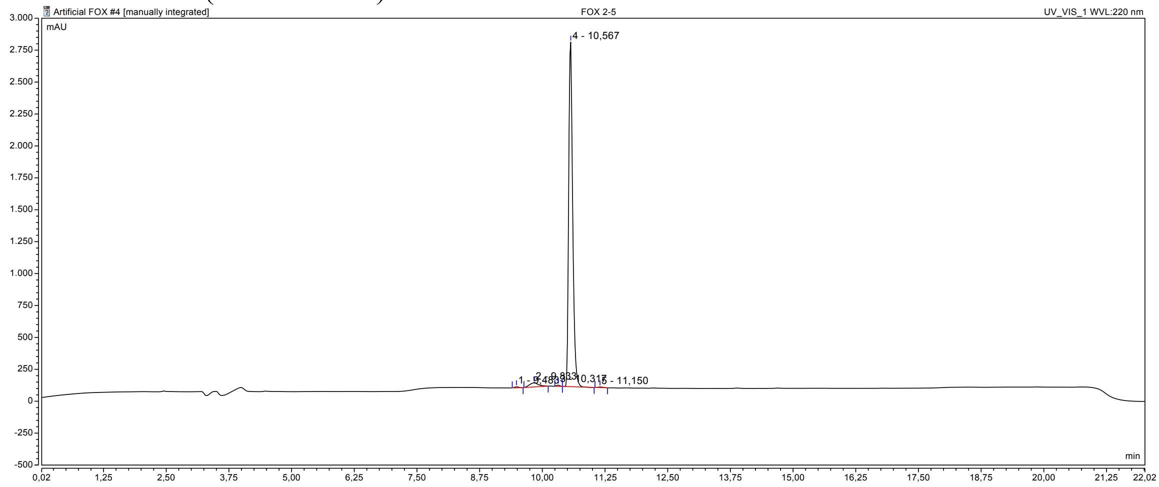

Name: **FOX 2-6**
Catalogue number: 0005121
Lot: JK-917
Mol. weight: 642.7 g/mol
Form: freeze-dried
Keep in the fridge
Purity: > 95% (by LCMS/NMR)

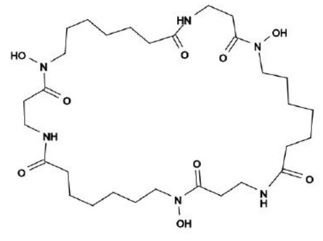

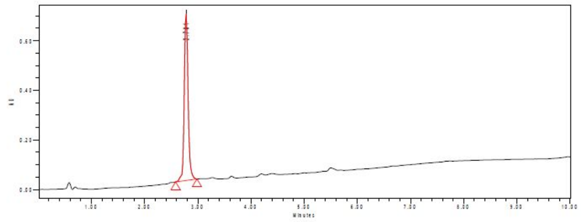

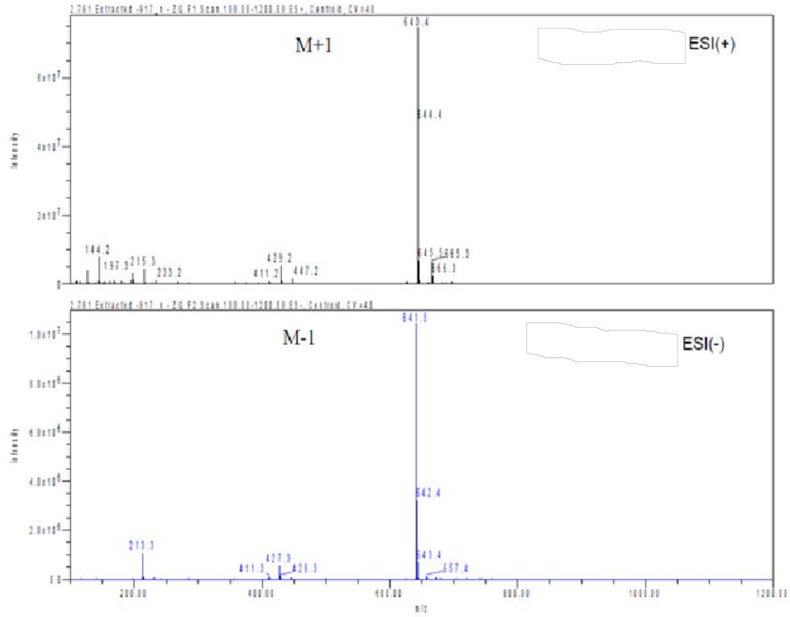

FOX 2-6: 95.1%(10-60% ACN)

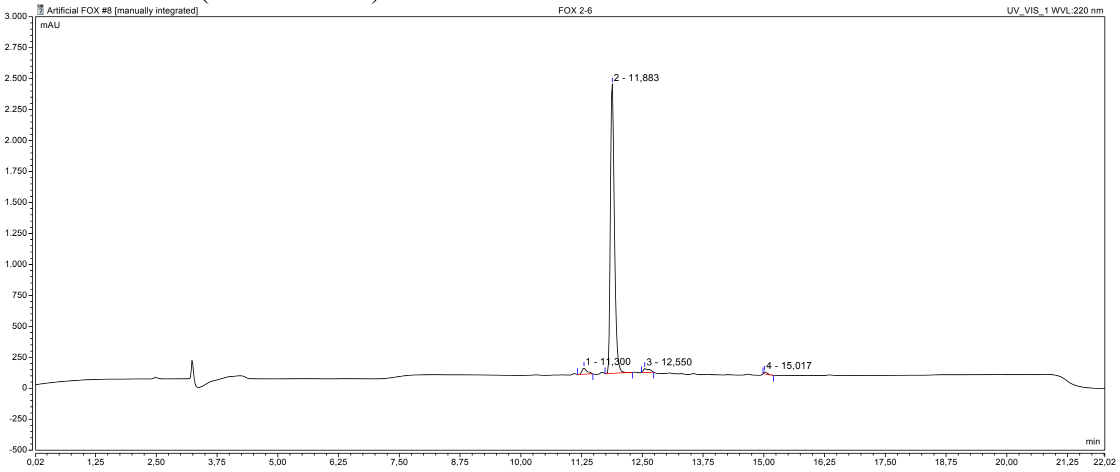

Name: **FOX 3-5**
Catalogue number: 0005122
Lot: JK-919
Mol. weight: 642.7 g/mol
Form: freeze-dried
Keep in the fridge
Purity: > 95% (by LCMS/NMR)

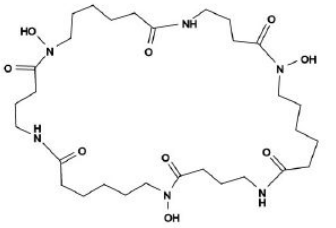

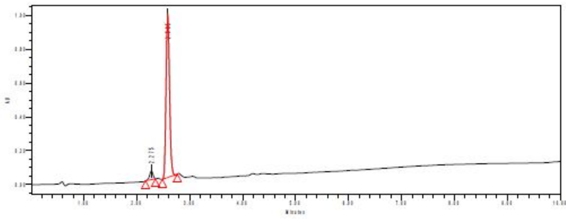

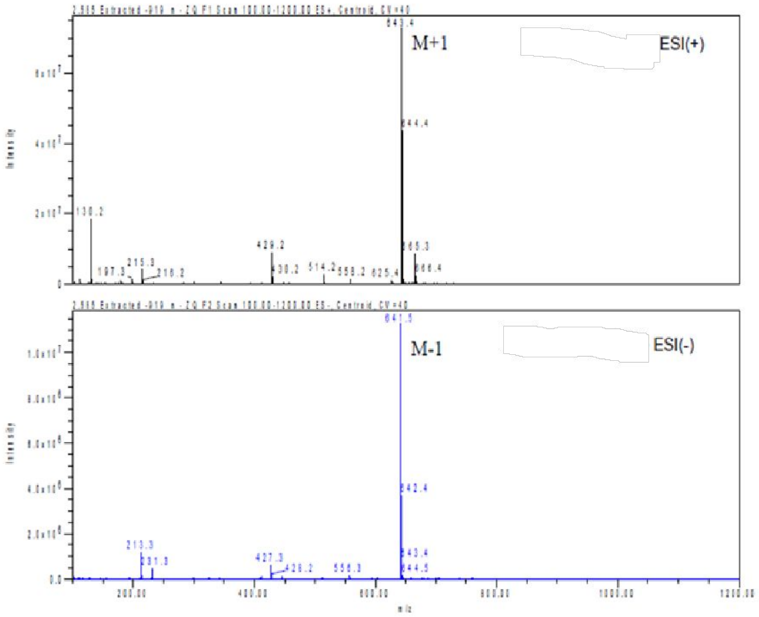

FOX 3-5: 96.9%(10-60% ACN)

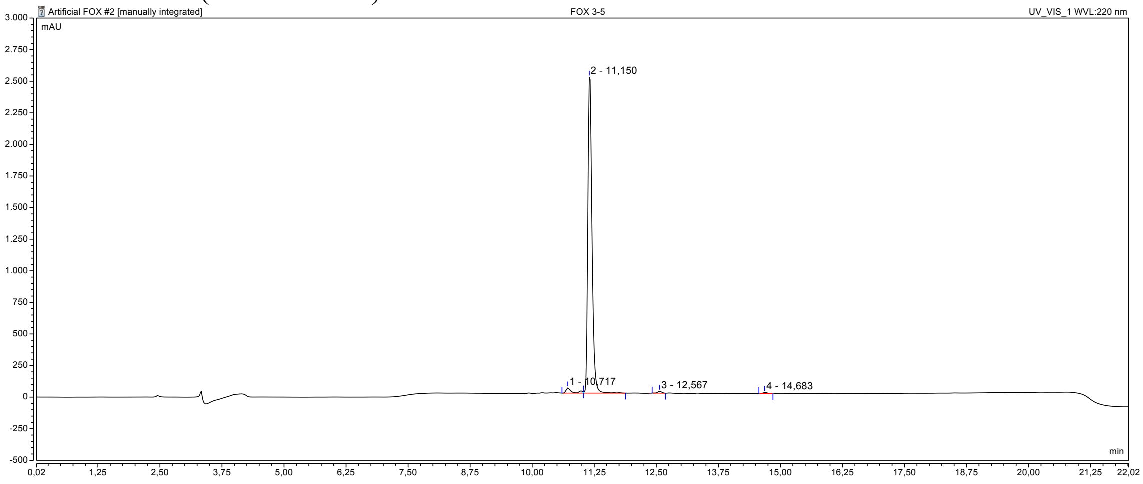

The purity of each compound was confirmed via potentiometric titrations.

2. Characterization of complexes

Characterization of Fe(III) and Ga(III) complexes of FOX analogs was determined in an elaborate studies involving ESI-MS, potentiometry and UV-Vis spectroscopy. Studied complexes were characterized by an exceptional stability constants and metal affinity within the range of natural siderophores. Summary of the coordination properties of the studied complexes are presented in the following section while full solution chemistry and thermodynamic characterization were described in our previous work. [1]

2.1 Coordination properties of studied ligands towards Fe(III) and Ga(III)

Table S1. Intensity maxima of the major Fe(III)-FOX and Ga(III) complexes and adduct ions observed by ESI-MS.

Fe(III)-FOX	Pseudomolecular ion	m/z experimental	m/z simulated
FOX 2-2	$\{[FeL] + [H]^{+}\}^{+}$	528.13	528.13
FOX 2-3	$\{[FeL] + [Na]^{+}\}^{+}$	592.15	592.16
	$\{[FeL] + [Na]^{+} + [NaClO_4]\}^{+}$	714.09	714.09
	$\{[FeL] + [Na]^{+} + 3[NaClO_4]\}^{+}$	957.95	957.97
FOX 2-4	$\{[FeL] + [Na]^{+}\}^{+}$	634.19	634.20
	$\{[FeL] + [Na]^{+} + [NaClO_4]\}^{+}$	756.13	756.14
	$\{[FeL] + [Na]^{+} + 3[NaClO_4]\}^{+}$	999.99	1000.01
FOX 2-5	$\{[FeL] + [Na]^{+}\}^{+}$	676.24	676.25
	$\{[FeL] + [Na]^{+}+[NaClO_4]\}^{+}$	798.17	798.19
FOX 3-5	$\{[FeL] + [Na]^{+}\}^{+}$	718.29	718.27
	$\{[FeL] + [K]^{+}\}^{+}$	712.26	734.27
	$\{[FeL] + [Na]^{+} + [NaClO_4]\}^{+}$	840.22	840.23
Ga(III)-FOX	Pseudomolecular ion	m/z experimental	m/z simulated
FOX 2-2	$\{[GaL] + [H]^{+}\}^{+}$	541.12	541.12
FOX 2-3	$\{[GaL] + [Na]^{+}\}^{+}$	605.14	605.14
	$\{[GaL] + [Na]^{+} + [NaClO_4]\}^{+}$	727.07	727.08
	$\{[GaL] + [Na]+ + 3[NaClO_4]\}+$	927.93	927.96
FOX 2-4	$\{[GaL] + [Na]^{+}\}^{+}$	647.19	647.19
	$\{[GaL] + [Na]^{+} + [NaClO_4]\}^{+}$	769.12	769.13
	$\{[GaL] + [Na]^{+} + 3[NaClO_4]\}^{+}$	1014.97	1015.00
FOX 2-5	$\{[GaL] + [Na]^{+}\}^{+}$	689.23	689.24
	$\{[GaL] + [Na]^{+} + [NaClO_4]\}^{+}$	811.16	811.18
	$\{[GaL] + [Na]^{+} + 3[NaClO_4]\}^{+}$	1057.02	1057.05
FOX 3-5	$\{[GaL] + [Na]^{+}\}^{+}$	731.28	731.29
	$\{[GaL] + [K]^{+}\}^{+}$	747.25	747.26
	$\{[GaL] + [Na]^{+} + [NaClO_4]\}^{+}$	853.21	853.22

Measured at conditions: [L]: 1□10^{-5} M M:L 1:1, MeOH/H_2O: 50/50 w/w.

Table S2. Overall stability constants ($log_{\beta}ML$) for studied complexes with Fe(III) and Ga(III) and pFe(III) and pGa(III) values.

Analog	$\log\beta_{Fe(III)L}$[a]	UV-VIS of Fe(III)L[b]	$\log\beta_{Ga(III)L}$[a]	pFe(III)[d]	pGa(III)[d]
FOX 2-2	25.92(8)	ε_{426} = 2730	26.44(5)	21.5	21.4
FOX 2-3	27.22(2)	ε_{432} = 2140	25.14(9)	22.7	20.8
FOX 2-4	28.71(7)	ε_{430} = 2450	26.29(7)	24.3	21.8
FOX 2-5	31.32(8)	ε_{440} = 2540	29.50(6)	27.0	25.2
FOX 3-5	28.81(2)	ε_{430} = 2660	27.17(4)	24.1	22.6
FOXE	32.21(4)[c]	ε_{435} = 2620	29.79(1)	27.3[c]	25.2

[a] Constants determined from potentiometric and UV−vis pH-dependent titrations. Conditions: [L] = 1×10^{-3} M, M:L 1:1 for potentiometric assays and 5□10^{-5} M, M:L 1:1 for UV−Vis assays, T = 25 °C, I = 0.1 M $NaClO_4$, [b] Determined under conditions: [L] = $5 \cdot 10^{-5}$ M, M:L 1:1, T = 25°C, I = 0.1 M $NaClO_4$. Experimental errors: λmax = ± 2nm, ε = ± 5%; units λ_{max} [nm], ε [$M^{-1}cm^{-1}$]. [d] Calculated for conditions: [L] = 1×10^{-4} [M] = 1×10^{-5} at pH 7.4, [c] Reference[2]

2.2 Radiolabeling

All ligands could be labeled with gallium-68 using 10 µg of the ligand at high molar radioactivity of 2-6 GBq/µmol. Quantitative labeling yields were determined by HPLC and TLC. Complexes with labeling yield below 95%, as determined by TLC, were purified by SPE before further use. Radiochemical purity (RCP) results are summarized in the following table.

Table S3. Radiochemical Purity (RCP) of [^{68}Ga]GaFOX analogs as determined by SPE, HPLC and TLC

Analog	SPE[a]			HPLC			TLC		
	n	RCP	sd	n	RCP	sd	n	RCP	sd
FOX 2-2	5	75.1	7.9	3	51.1	12.3	5	13.8	5.4
FOX 2-3	5	52.5	6.8	3	13.4	4.1	5	13.8	5.4
FOX 2-4	5	94.2	2.3	5	83.4	2.4	5	15.1	8.8
FOX 2-5	n.d.	n.d.	n.d.	5	99.3	0.4	5	99.1	0.9
FOX 2-6	3	>90[b]		4	90.6	5.9	4	90.2	8.0
FOX 3-5	5	86.5	24.3	3	80.8	3.1	5	n.a.	n.a

[a]RCP calculated as percentage of bound (eluted in the ethanol fraction) over total activity eluted; [b]No exact calculation was made, as in one case RCP exceeded 100% due to geometry effects; n.d.: not determined (as RCP was exceeding 95% in other analysis), n.a.: not applicable due to insufficient separation

References:

1. Mular, A.; Shanzer, A.; Kozlowski, H.; Hubmann, I.; Misslinger, M.; Krzywik, J.; Decristoforo, C.; Gumienna-Kontecka, E. Cyclic analogs of desferrioxamine E siderophore for Ga-68 nuclear imaging: Coordination chemistry and biological activity in *Staphylococcus aureus*. *Inorganic Chemistry* **2021**, *60* (23), 17846-17857. DOI: 10.1021/acs.inorgchem.1c02453.

2. Konetschnyrapp, S.; Jung, G.; Raymond, K. N.; Meiwes, J.; Zahner, H. Solution thermodynamics of the ferric complexes of new desferrioxamine siderophores obtained by directed fermentation. *Journal of the American Chemical Society* **1992**, *114* (6), 2224-2230. DOI: 10.1021/ja00032a043.
